# Ultrahigh strength magnesium via solidification of nanocolloid

**DOI:** 10.1038/s41467-026-71671-x

**Published:** 2026-04-10

**Authors:** Xinliang Yang, Hari Babu Nadendla, Changming Fang, Shunsuke Nishi, Tomoki Matsuda, Makoto Kambara, Toshimi Tanaka, Masashi Dougakiuchi, Fengzai Tang, Geoffrey D. West, Shihao Wang, Quentin M. Ramasse

**Affiliations:** 1 BCAST, Brunel University of London, Kingston Lane, Uxbridge, UK; 2 Division of Materials and Manufacturing Science, Graduate School of Engineering, The University of Osaka, 1-1 Yamadaoka, Suita, Osaka Japan; 3 Takeuchi Electric, 51-1, Hokuryo-cho, Matsue, Shimane Japan; 4 Shimane Institute for Industrial Technology, 1 Hokuryo-cho, Matsue, Shimane Japan; 5 WMG, University of Warwick, Coventry, UK; 6 SuperSTEM Laboratory, SciTech Daresbury Science and Innovation Campus, Keckwick Lane, Daresbury, UK; 7 School of Chemical and Process Engineering, University of Leeds, Leeds, UK; 8 School of Physics and Astronomy, University of Leeds, Leeds, UK

**Keywords:** Structural properties, Mechanical engineering, Structure of solids and liquids, Metals and alloys

## Abstract

We report a simple, scalable route to produce ultrahigh-strength magnesium (Mg) via solidification of a colloidal solution containing nanoscale niobium carbide (NbC) particles suspended in liquid magnesium (Mg(l)). A single-atom-level investigation reveals that NbC exhibits spontaneous wetting with molten Mg, driven by the formation of an ordered layer of Mg atoms strongly bonded to the carbon atoms on the NbC {001} surface. This creates Mg-coated NbC (Mg@NbC) particles in liquid Mg and is referred to as Mg(l)-Mg@NbC nanocolloid. This unique and spontaneous wetting behaviour enables uniform nanoparticle dispersion in the molten Mg without external fields, and in the solidified Mg matrix without the need for thermomechanical processing. The resulting NbC dispersoids act as coherent, hard reinforcement phases, significantly strengthening the Mg matrix. As a result, the Mg-NbC material exhibits ultrahigh tensile strength and stiffness, surpassing those of all previously reported Mg alloy systems.

## Abstract (web summary)

A solidification strategy enables stable ceramic nanoparticle dispersion in molten Mg via an atomically bonded interface, ensuring exceptional wetting and uniform incorporation in casting, overcoming long-standing barriers in metal–ceramic systems.

## Introduction

Mg has a low density of 1.78 g·cm^−3^ and a high damping capacity with a loss factor ~11.8 × 10^−3^ (~10 times higher than Al and Steel), and thus offers tremendous potential in achieving lightweight vehicles with improved fuel economy and reduced CO_2_ emission. However, the yield strengths of the most used automotive Mg alloys are in the range of 100–200 MPa, which is just nearly half that of Al alloys and 1/4 that of high strength steels; thus, the industry is not fully able to utilise their lightweight potential.

Among the major alloy strengthening mechanisms, the uniform dispersion of nanoscale secondary phase precipitates is particularly effective^1^. Alloying additions of rare earth (RE) elements in Mg alloys results in a significant strength improvement due to the effective precipitation of RE-containing phases upon heat treatment^2,3^. Despite the promise of this approach, the high cost and limited availability of RE elements have historically hindered the widespread use of Mg-RE alloys in industry, with their potential only recently being actively explored^4,5^.

As another strategy, fine-grained magnesium produced through thermomechanical processing is typically combined with precipitation hardening to achieve high strength^6^. However, the resulting microstructures often exhibit limited thermal stability, which compromises their performance under demanding service conditions.

Ceramic particles with high shear moduli, as an alternative approach to the in-situ formed precipitates, can serve as the non-deforming particles that favour the Orowan strengthening mechanism^7^. Their strengthening effect depends mainly on particle size and number density. This approach does not require specially tailored alloy compositions or complex thermomechanical processing routes. Reported data on various metallic systems highlight that in addition to ceramic particles contributing to strengthening^8–12^, the resulting nano-sized grain structure of the metallic matrix significantly improves the overall mechanical strength. For example, in Mg alloys, a multi-step external field treatment on molten metal followed by severe plastic deformation was conducted to disperse SiC nanoparticles^13^. In molybdenum alloys, the in-situ reaction of the precursor compounds^14^ was utilised to introduce the nano-sized ceramic particles, and then a rolling process was conducted to disperse the nanoparticles and refine the Mo grain size. These ceramic phases are thermally stable and can effectively retain their strengthening effect at elevated temperatures. This is the case of Al incorporated with graphene-coated MgO nanoparticles^9^, via the powder metallurgy route, which showed improved strength and creep resistance at temperatures as high as 500 °C. Similarly, non-equilibrium solidification in additive manufacturing was applied to incorporate La_2_O_3_ nanoparticles in a medium entropy alloy matrix and demonstrated further enhancement of creep property at 1093 °C^15^ compared with the pure alloy parent phase.

We report here an ultrahigh tensile strength of ~680 MPa in Mg produced by solidification of a Mg colloid containing fine scale NbC dispersions. The favourable wetting characteristics of the NbC phase with molten magnesium enabled the processing of such high-strength material. We investigate the fundamental mechanism for the atomic interaction between Mg and NbC using aberration-corrected scanning transmission electron microscopy (STEM) and electron energy loss spectroscopy (EELS). We further perform density functional theory (DFT) calculations to investigate the interfacial interactions and atomic arrangements at the liquid Mg/NbC (Mg(l)/NbC) interfaces, with an emphasis on the non-polar NbC {001} surface that terminates most produced NbC nanoparticles. This study reveals that the formation of an ordered layer of Mg atoms, strongly bonded to the carbon atoms on the NbC {001} surface, results in Mg-coated NbC (Mg@NbC) colloid, which plays a critical role in the observed favourable wetting characteristics. During solidification of the Mg(l)-Mg@NbC colloid, the Mg solidification front spontaneously engulfs the NbC dispersoids into the growing Mg grains. In the resulting solidified alloys, these embedded dispersoids can effectively contribute to strengthening, similar to the role of coherent precipitates in monolithic Mg alloys.

## Results

### Mg-NbC material by solidification of colloidal solution

Historically, the preparation of stable high-temperature colloidal solutions via the combination of molten metal and ceramic particulate dispersions has not been possible, due to the poor wettability of the ceramic particles by the molten metal^16^. We have identified the metal-ceramic (Mg-NbC) system as a promising candidate to realise this task, owing to its favourable wetting characteristics that enable the production of a high-temperature nanocolloidal solution consisting of NbC nano-dispersions in molten Mg using a simple, practical and scalable method.

**Fig. 1:**
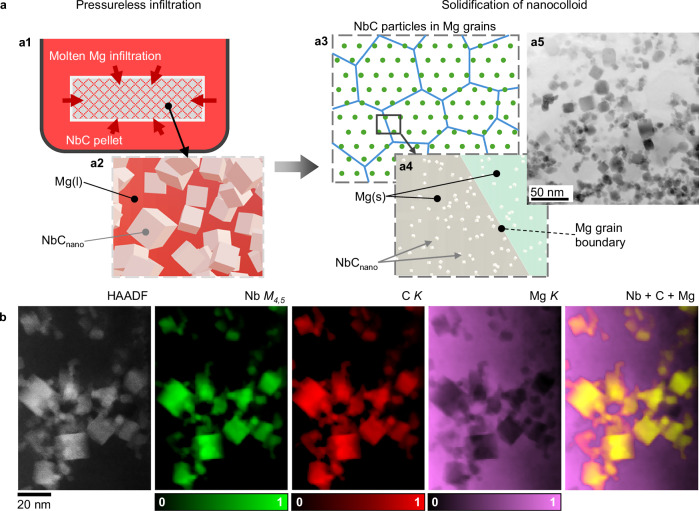
Preparation of Mg-NbC sample by nanocolloid solidification. **a** Schematic diagram of the material preparation (**a**1 molten Mg infiltration into packed NbC powders, **a**2 Mg(l)-Mg@NbC nanocolloid, **a**3 microstructure of the solidified nanocolloid, and **a**4 illustration of NbC_nano_ particles engulfed within the Mg grains (grey and green colours represent randomly oriented Mg grains)). **a**5 Bright-field TEM image revealing a uniform dispersion of nanoparticles in the solidified Mg-NbC_nano_. **b** High-angle annular dark-field (HAADF) STEM image and the corresponding simultaneously acquired EELS maps for Nb, C, Mg and false-colour composite, confirming the presence of the Mg phase in between NbC nanoparticles.

For producing nanocolloidal solutions, we first compressed NbC powders with particle size of 15 nm (NbC_nano_), 287 nm (NbC_submicron_) and 906 nm (NbC_micron_) (see “Methods”) into bulk pellets and then infiltrated molten Mg into these compressed pellets at ambient pressure (Fig. 1). The colloidal solution was then solidified using a conventional casting process to obtain the Mg-NbC material that consists of uniform NbC nanodispersoids in the Mg matrix (Supplementary Figs. 1, 2 for the case of NbC_nano_ particles). During infiltration into the compressed powder pellet, molten Mg wetted the nanoparticles causing them to detach from each other and form a stable nanocolloid. This preparation method significantly simplifies the incorporation of ex-situ ceramic nanoparticles into the molten metal with a uniform dispersion. Similarly, uniform dispersions can also be seen in Mg-NbC_submicron_ and Mg-NbC_micron_ materials prepared using this approach (Supplementary Figs. 3–6). It is worth noting that, to our knowledge, the favourable wetting behaviour observed in the Mg-NbC system has not been reported in structural engineering metal/ex-situ ceramic systems with nano-sized ceramic particles. To exploit this favourable wetting behaviour and to better emulate industrial processing routes for producing larger volumes of Mg-NbC materials, the infiltrated Mg-NbC_submicron_ pellet was introduced into a molten Mg bath (Supplementary Fig. 7). This process successfully yielded bulk Mg containing 1 vol.% NbC_submicron_ particles.

### Ultrahigh tensile strength in Mg-NbC materials

**Fig. 2:**
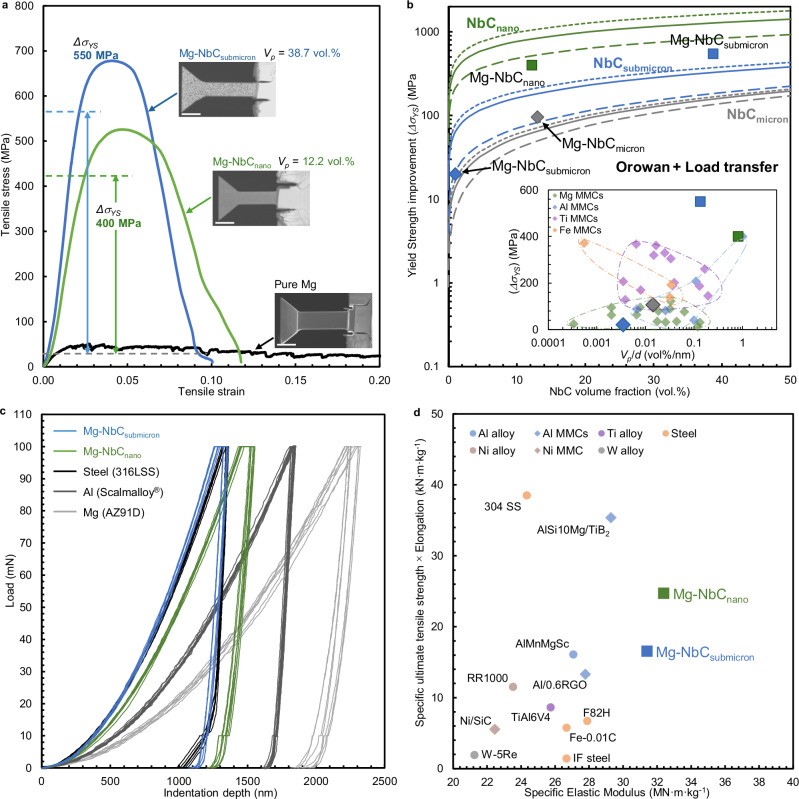
Mechanical properties of as-solidified Mg-NbC materials. **a** Tensile stress-strain curves of as-solidified Mg-NbC_submicron_, Mg-NbC_nano_ and Mg obtained from micro-tensile tests. The improvement in yield stress is marked and the insets are the images of the corresponding micro-tensile samples. Scale bars, 5 μm. **b** Calculated and experimentally measured yield strength improvement ($\Delta {\sigma }_{{YS}}=\Delta {\sigma }_{{YS}({Mg}-{NbC})}-\Delta {\sigma }_{{YS}({Mg})}$) of the Mg-NbC materials containing three different particle sizes. The curves represent calculated values, given by the sum of Orowan strengthening ($\Delta {\sigma }_{{Orowan}}$) and load-transfer strengthening ($\Delta {\sigma }_{{\rm{LT}}}$), as a function of NbC volume fraction (*V*_*p*_) for micron, submicron and nanoscale particles. The square and diamond symbols represent micro-tensile and macro-tensile test results. The dotted, solid and dashed lines correspond to the calculation using particle sizes of *d*_peak-σ_, *d*_peak_, and *d*_peak+σ_, respectively. The inset shows $\Delta {\sigma }_{{YS}}$ for various composites processed by different methods (powder metallurgy, additive manufacturing and melt casting) as a function of *V*_*p*_*/d*, where d is the size of reinforcement. The data for the Mg-NbC materials presented in the inset share the same symbol codes as those in panel (**b**). The green, blue, purple and orange envelopes correspond to Mg MMCs^s7-s20^, Al MMCs^9,s21-s24^, Ti MMCs^s25-s37^, and Fe MMCs^s38-s40^, respectively. A significant improvement in tensile yield strength is observed in Mg-NbC materials processed using a simple melt processing route. **c** The indentation load-depth curves of Mg-NbC with benchmark engineering alloys (AZ91D alloy (Mg), AlMgSc Scalmalloy^®^ (Al), and 316 L stainless steel). The Mg-NbC_submicron_ shows a higher indent resistance than the engineering Mg, Al and Steel alloys. **d** The product of specific ultimate tensile strength and elongation against the specific elastic modulus of as-solidified Mg-NbC_nano_ stands out, in comparison with the values for other high performance metallic materials and their metal matrix composites reported in the literature. The blue, purple, orange, coral and grey circular symbols correspond to Al alloy^55^, Ti alloy^56^, steels^57–60^, Ni superalloy^61^, and tungsten alloy^62^, respectively. The blue and coral diamond symbols correspond to Al MMCs^63,64^ and Ni MMC^65^, respectively.

The measured tensile strength of Mg-NbC materials (Fig. 2a) prepared by solidification of Mg(l)-Mg@NbC colloid is over one order of magnitude higher than pure Mg. For Mg-NbC_nano_ and Mg-NbC_submicron_ materials, the measured strengths are 527 MPa (~10 times higher) and 678 MPa (~14 times higher), respectively. Even with such high load of ceramic particles, the tested Mg-NbC samples exhibited a ductile fracture morphology (Supplementary Fig. 8). To further evaluate the influence of particles with various size and volume fraction, the experimental data were compared with theoretical predictions in Fig. 2b. Orowan strengthening^7,13^ and load-transfer strengthening^17^ are considered as the dominant strengthening mechanisms (see “Methods”). The solid-solution strengthening is absent in these materials as the matrix is pure Mg. In the absence of grain boundaries in Mg-NbC_nano_ and Mg-NbC_submicron_ materials (Supplementary Table 1), grain boundary strengthening is also excluded. However, due to polycrystalline nature of macro-tensile samples (both Mg-1vol.%NbC_submicorn_ and Mg-13vol.%NbC_micron_), the grain boundary strengthening is considered (see “Methods”). For comparison, reported yield strength improvement ($\triangle {\sigma }_{{YS}}$) for a range of materials containing ex-situ phase additions (Supplementary Table 2) are compiled and shown in the inset of Fig. 2b alongside the Mg-NbC_nano_ and Mg-NbC_submicron_ samples. As shown, the Mg-NbC system exhibits significantly higher $\triangle {\sigma }_{{YS}}$ values compared to Mg, Al, Ti, and Fe based metal matrix composites (MMCs).

The indentation test results (Fig. 2c) show a high hardness for Mg-NbC_submicron_ (2.59 GPa), exceeding that of 316 L stainless steel (2.45 GPa), thought to be due to the presence of hard NbC dispersions in the metal matrix. Higher specific elastic modulus and hardness for all Mg-NbC samples over other engineering alloys are also achieved (Supplementary Fig. 9).

The mechanical properties of a range of metallic materials and MMCs, measured using the micro-tensile method and reported in the literature, are tabulated in Supplementary Table 3. The product of specific ultimate tensile strength and elongation (which is an indicator for specific toughness) is plotted against the specific elastic modulus in Fig. 2d. Mg-NbC_nano_ exhibits a unique combination of specific elastic modulus (~32 MN·m·kg^−1^) and specific toughness indicator (~25 kN·m·kg^−1^). This demonstrates the advantages of this ex-situ particle strengthening strategy for simultaneous enhancement of specific stiffness and toughness at room temperature. While these metrics clearly highlight the performance advantages of Mg-NbC, the broader applicability of this approach for advanced structural applications will ultimately depend on its high-temperature mechanical response and microstructural stability; comprehensive investigations in this regard are currently ongoing and will be reported in a future dedicated publication.

### Mg/NbC interfaces at atomic scale

To understand the wetting behaviour of NbC by molten Mg, as well as the origin of the uniform nanoparticle dispersion and the resulting mechanical performance observed in the Mg-NbC system, we now turn to a detailed investigation of the Mg/NbC interface. First, we present atomic-scale experimental evidence using scanning transmission electron microscopy (STEM) combined with electron energy-loss spectroscopy (EELS), which reveals the presence of a thin, ordered layer of Mg atoms on the surface of the NbC particles. Next, we support and extend these observations through first-principles density functional theory (DFT) simulations, which provide insights into the atomic bonding and interfacial energetics responsible for this unique surface structure.

**Fig. 3:**
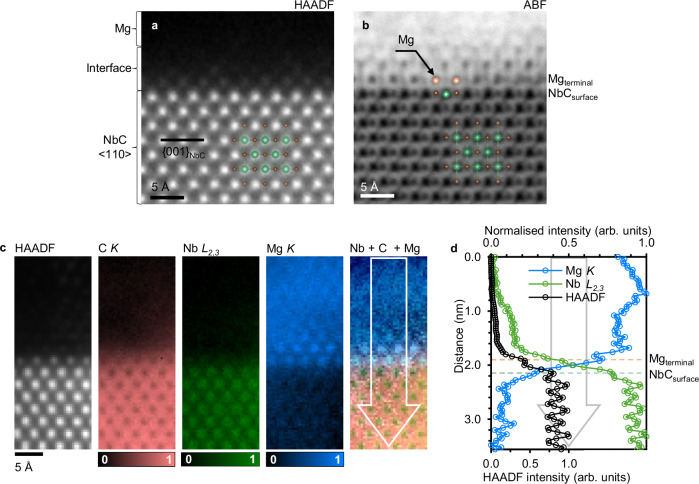
Atomic resolution characterisation of structure and composition across the Mg/NbC_{001}_ interface by STEM imaging and EELS mapping. **a** HAADF and (**b**) annular bright-field (ABF) images of the interface. **c** HAADF survey image and the simultaneously acquired EELS maps for C, Nb, Mg and a composite elemental superposition, and **d** Compositional and HAADF-contrast line profiles in arbitrary units (arb. units) plotted along the white arrow in (**c**) with intensity values averaged over the arrow’s width. (Dashed lines indicate NbC_surface_ and Mg_terminal_, as a guide to the eye). For all the STEM images and EELS maps, the incident beam is parallel to the zone axis <110>_NbC_. Schematic atomic configurations are overlaid on the images in (**a**, **b**) in which green, dark brown and orange spheres represent Nb, C and Mg atoms, respectively. The compositional line profiles across the interface (averaged over the width of the arrow overlaid on the composite colour map) confirms the formation of an ordered Mg multilayer on the NbC surface.

As the introduction of nanoparticles into molten metal is the most challenging, and their strengthening effectiveness is superior among the three Mg-NbC materials synthesised and studied here, the Mg-NbC_nano_ material has been selected for in-depth interfacial analysis. We employed dedicated STEM imaging combined with EELS core-loss mapping to simultaneously resolve the Mg/NbC_{001}_ interfacial structure and its chemical composition. The images in Fig. 3a, b provide complementary contrast sensitive to the interfacial atomic structure^18,19^, while the corresponding compositional distribution is further resolved by EELS elemental mapping in Fig. 3c. We can readily recognise interfacial atomic layers of different characteristics to the NbC substrate, which are confirmed to be rich in Mg. Predominant atomic features in each layer can be distinguished by the relative compositions across the interface (Fig. 3c, d). Notably, this interfacial multilayer complexion is epitaxially coherent with the NbC {001} plane and each Mg atomic column in the terminating Mg layer is seen to coordinate with a C atomic column immediately beneath it. This interfacial Mg atomic arrangement is referred to as C_NbC_-like arrangement. As the distance away from the NbC {001} surface increases, the Mg atoms arrangement deviates from the C_NbC_-like configuration and adopts a hexagonal close packed (HCP) structure (Supplementary Fig. 10).

**Fig. 4:**
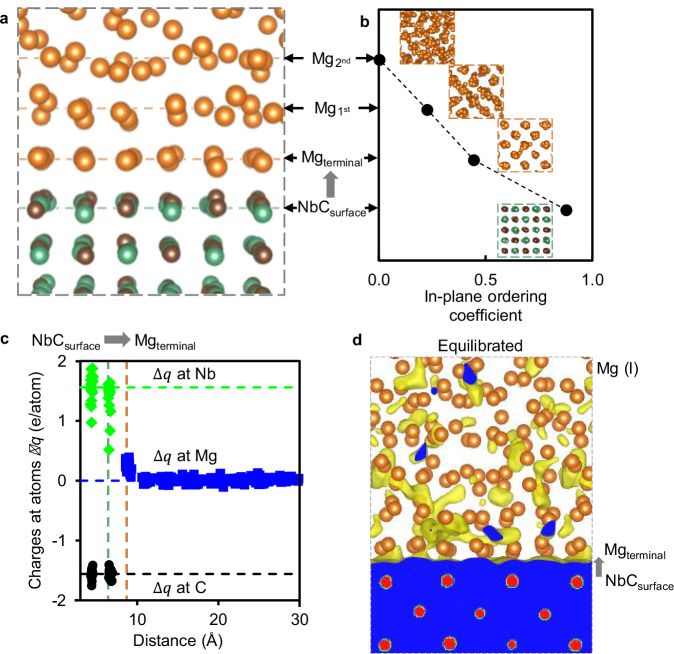
DFT analysis of the Mg(l)-NbC. **a** A snapshot of the atomic arrangements of Mg(l)/NbC_{001}_ interface equilibrated at 1000 K. Green, brown and orange spheres represent Nb, C and Mg atoms, respectively. Dashed lines indicate individual atomic layers. **b** In-plane ordering coefficient at the interfaces for configurations summed over 3 ps. Insets are the atomic arrangement of the Nb and C atoms at the outmost substrate layer, Mg atoms at the terminal layer (Mg_terminal_), Mg atoms at 1^st^ layer (Mg_1st_) and 2^nd^ layer (Mg_2nd_), Strong ordering is seen within the Mg_terminal_ atomic layer. Then the ordering coefficient rapidly reduces for Mg_2nd_ atomic layer which is typical for liquid-like structures. **c** The Bader charges at atomic sites at the Mg(l)/NbC_{001}_ interface in which the black, blue and green data points represent net charges at C, Mg and Nb sites, respectively. Dashed green and orange vertical lines represent the NbC_surface_ atomic layer and Mg_terminal_ atomic layer, respectively. Dotted black and green horizontal lines represent charge values at C and Nb, respectively, in bulk NbC. **d** Iso-surfaces of electron density distribution (*ρ*_*0*_*(****r****)* = 0.018e/Å^3^). The yellow clouds represent the iso-surfaces with *ρ*_*0*_*(****r****)* = 0.018e/Å^3^. Blue and white coloured region represents higher and lower electron density, respectively. The red spherical clouds in the substrate originate from the Nb 4 d electrons.

First-principles DFT simulation results for the liquid Mg (Mg(l)) and NbC solid substrate system are shown in Fig.4. A snapshot of the atomic arrangements of the Mg(l)/NbC_{001}_ interface (viewed along the <100>_NbC_ direction) equilibrated at 1000 K is presented in Fig. 4a. The corresponding atomic arrangement along the <110>_NbC_ direction is provided for completeness in Supplementary Fig. 11. Strikingly, the simulated interfacial structure shows feature similar to those observed in the post-mortem Mg-NbC_nano_ material after solidification (Fig. 3). The Nb and C atoms in the substrate are well-ordered and the substrate remains solid at 1000 K. The Mg atoms in the melt adjacent to the substrate (terminating Mg atoms) exhibit a layering phenomenon with a high degree of solid-like atomic ordering^20^ as shown in the atomic density profile (Supplementary Fig. 12). This ordering is quantified using the in-plane ordering coefficient, which is calculated as the average of local order parameters of the atoms within the individual layer at the interface^21^. Our calculations show that the Mg coating layer has an average in-plane ordering coefficient of 0.45, approximately half the value observed for the adjacent solid NbC layer. Each surface C atom is coordinated with one terminating Mg atom. Those terminating Mg atoms exhibit ordered structures similar to that of C atoms in face centred cubic NbC (C_NbC_-like arrangement). The C-Mg interatomic distances vary with typical lengths between 2.0 Å to 2.4 Å (Supplementary Fig. 13). The in-plane ordering coefficient rapidly decreases with increasing distance from the NbC substrate to the Mg atomic layers considered. The Mg atoms within the second layer have weak long-range ordering; this region is more liquid-like and correspondingly the in-plane ordering coefficient is just 0.04 (Fig. 4b).

The calculated net charge distribution at the Mg(l)/NbC_{001}_ interface is shown in Fig. 4c. The C atoms at the NbC surface layer exhibit the same valence as in the bulk (−1.56 e^−^/C). Meanwhile, surface Nb atoms lose 1.37 e^−^/Nb on average, which is slightly less than that in the bulk (1.56 e^−^/Nb). The Mg_terminal_ atoms lose 0.2 e^−^/Mg on average, which indicates a moderate charge transfer to the coordinating C atoms beneath. The iso-surfaces of electron density distribution at the Mg(l)/NbC_{001}_ interfacial area in Fig. 4d show a high electron density (blue shade) within the NbC substrate. The Mg atoms that are bonded to C have low electron density around them, suggesting an ionic nature^22^ of the bonds, which promoted the localised C_NbC_-like arrangement of Mg atoms adjacent to the NbC substrate. In contrast, the Mg atoms away from the interface consists of a free-electron cloud and ‘naked’ Mg ions.

Overall, the DFT calculations confirm the formation of an ordered layer of Mg atoms being strongly bonded to the surface-terminating C atoms, forming a Mg-coated NbC surface (Mg@NbC).

The combined experimental observations using atomic-resolution STEM-EELS in the as-solidified Mg-NbC_nano_ sample (Fig. 3) with supporting DFT calculations (Fig. 4) unravel a C_NbC_-like arrangement of the ordered Mg layer on the NbC substrate. This suggests that Mg atoms spontaneously adhere to the surface of the NbC phase, as shown in Supplementary Fig. 12, driven by chemical attraction between NbC and Mg(l). Such interfacial adherence results in enhanced macroscopic wetting. During solidification, the C_NbC_-like arrangement of the terminal Mg layer remains stable due to chemical bonding between Mg and C atoms on NbC particles. A typical hcp structure of Mg forms beyond this ordered Mg multilayer, as observed in the solidified sample (Supplementary Fig. 10).

### Stable Mg-NbC nanocolloid at high temperature

To achieve a stable nanocolloid in a molten magnesium medium (1000 K) with NbC nanoparticles of 15 nm diameter, the following conditions must be satisfied:

(i) The thermal energy associated with nanoparticle Brownian motion (*E*_*b*_) must exceed the van der Waals attraction energy (*W*_*vdWaals*_) between particles, ensuring dispersion rather than aggregation.
(ii) The energy barrier that prevents nanoparticle contact and sintering (*W*_*barrier*_) must be greater than the Brownian motion energy (*E*_*b*_), so that random collisions do not lead to irreversible bonding or clustering.

Thermal energy for nanoparticle dispersion: *E*_*b*_ is calculated to be 13.8 zJ (*E*_*b*_ = *kT*, where *k* is the Boltzmann constant and *T* is the processing temperature of 1000 K).

Van der Waals attraction: The van der Waals interaction between two naked or coated NbC nanoparticles in molten Mg can be estimated by the equation^23,24^:

1 $${W}_{{vdWaals}}=-\frac{{A}_{{NbC}-{Mg}\left(l\right)-{NbC}}}{6D}\cdot \frac{{R}_{{NbC}}}{2}$$

2 $${A}_{{NbC}-{Mg}\left(l\right)-{NbC}}\approx {\left(\sqrt{{A}_{{NbC}}}-\sqrt{{A}_{{Mg}(l)}}\right)}^{2}$$

where *D*, the minimum distance between two nanoparticles, is 0.2 nm (atomic layer of liquid Mg^25^). *R*_*NbC*_, the average radius of NbC nanoparticles, is 7.5 nm. *A*_*Mg(l)*_ and *A*_*NbC*_ are the Hamaker constants of molten Mg and naked NbC for the van der Waals interaction. *A*_*Mg(l)*_ is 206zJ for molten Mg^26^ and *A*_*NbC*_ is calculated to be 222 zJ using Lifshitz theory^27,28^. Therefore, the absolute value of the maximum van der Waals attraction (*W*_*vdWaals*_) between two naked NbC nanoparticles in liquid Mg is estimated to be 0.94 zJ, which is less than the thermal energy for Brownian motion.

In the case of Mg-coated NbC particles (Mg@NbC), Prieve and Russel^27^ have demonstrated that coating can mask the substrate particle and alter the Hamaker constant of the substrate particles towards the value of the coating phase (here Mg phase) when the distance is very close (*D* → 0). Thus, the value of *A*_*NbC-Mg(l)-NbC*_ in Eq. (2) will be decreased significantly between Mg@NbC particles in the molten Mg medium. Therefore, for Mg coated NbC particles in the molten Mg media, the Brownian motion dominates, and the particles will be unlikely to aggregate.

Energy barrier preventing naked nanoparticle contacting: At high temperatures, nanoparticles may sinter together upon contact, driven by a reduction in interfacial energy. In a Mg(l)-Mg@NbC colloid, as shown in Fig. 3a, a stable C_NbC_-like ordered Mg layer forms facilitated by ionic bonding between Mg and C on the outermost NbC {001} surface. For NbC_nano_ particles to make contact and sinter within the molten Mg, the Mg-C bonds at the Mg(l)/NbC_{001}_ interface must be broken. The bonding energy of Mg-C is estimated using MgC in a rock salt-type structure^29^ due to its similar Mg-C bond lengths of 2.0–2.5 Å. The bonding energy^30^ is defined as *ΔE* = *E*_*(Mg-C)*_ *- n·E*_*(Mg-Mg)*_, where *E*_*(Mg-C)*_ and *E*_*(Mg-Mg)*_ represent the bond energies of a single Mg-C bond in MgC and a single Mg-Mg bond in hcp Mg, respectively; *n* is the quantity of lost Mg-Mg bonds due to the formation of Mg-C bond for the Mg atoms adjacent to the NbC substrate. The calculated average interfacial bonding energy for Mg-C is −1.54 J·m^−^².

The debonding work (*W*_*debonding*_) required for NbC_nano_ particles to come into contact and sinter is given by *W*_*debonding*_ = *S*·*σ*_*Mg*−*C*_, where *S* is the effective area of interaction (calculated as *S* = *πRD*_*0*_ with *R* = 7.5 nm, and *D*_*0*_ = 0.2 nm^25^) and ${\sigma }_{{Mg}-C}$ is the interfacial bonding energy, −1.54 J·m^−^². The resulting debonding work is calculated to be 7.25 × 10^3^ zJ, which is approximately three orders of magnitude higher than the thermal energy (*E*_*b*_ = 13.8 zJ). Consequently, the nanoparticles are unlikely to contact and sinter with one another under these conditions.

In brief, once naked NbC particles are introduced into liquid Mg, the formation of a Mg-coated layer on NbC leads to a stable Mg(l)-Mg@NbC nanocolloidal solution. Colloid stability for larger particles with influence of gravity is detailed in Supplementary Note 1.

### NbC particle engulfment by the Mg solidification front

In typical solidification processes, ceramic particles (especially at the submicron or nanoscale) are often rejected at the solid/liquid interface due to interfacial energy considerations. This leads to particle segregation and clustering along grain boundaries, which can severely degrade the mechanical performance of the solidified metal. In contrast, as shown in Supplementary Figs. 2b, 4c, 6c, the Mg-NbC system surprisingly exhibited uniform particle dispersions with a wide range of particle sizes (from nanoscale to microscale) in the entire solidified Mg matrix, indicating a favourable interfacial interaction. This characteristic is critical for maintaining the uniform dispersion of nanoparticles initially achieved in the nanocolloidal solution and is a key factor in achieving the observed ultrahigh mechanical performance. Kaptay^31^ has proposed an interfacial criterion for spontaneous and forced engulfment of ceramic particles by an advancing solid/liquid interface. The sign of the interfacial force acting between a ceramic particle (c) and a solidification front (s) through the thin layer of a liquid (l) is determined by the sign of the quantity $\Delta {\sigma }_{{cls}}$ (*Δσ*_*cls*_ *= σ*_*cv*_ *- σ*_*lv*_ *× (*0.08 + 1.22 *× cosΘ*_*clv*_*)* where *σ*_*cv*_ is the surface energy of the ceramic, *σ*_*lv*_ is the surface tension of molten metal and *Θ*_*clv*_ is the contact angle of the molten metal on the ceramic substrate). The interfacial force is attractive, i.e., spontaneous engulfment of reinforcing particles by the solidification front is expected, if $\Delta {\sigma }_{{cls}} < 0$. Kaptay^31^ applied this criterion to a range of Al-ceramic systems (TiC, SiC, Al_2_O_3_, SiO_2_ and TiB_2_) and found that the *Δσ*_*cls*_ values are positive for systems in which ceramic particles were observed to be pushed by the solidification front leading to particle segregation at the inter-grain area, which is undesirable for strength enhancement.

Due to the formation of an ordered C_NbC_-like Mg layer on the exposed surfaces of NbC particles, the effective interface between the liquid Mg and particle (Mg(l)/NbC) becomes Mg(l)/Mg_terminal_ at Mg@NbC. This implies a favoured wetting of the NbC substrate by the molten Mg with a contact angle approaching zero (*Θ*_*clv*_ = 0). As a result, during solidification, the advancing Mg front interacts with the Mg@NbC particle to form a Mg(s)/Mg_terminal_ interface rather than a direct Mg(s)/NbC interface. As shown in Fig. 3a and Supplementary Fig. 13c, each terminal Mg atom exhibits 12 neighbours (four Mg atoms within the same plane, three Mg atoms in the 1^st^ Mg layer, four Nb atoms and one C in the underlying NbC layer). Although crystallographically distinct, this face-centred-cubic-like coordination provides each Mg atom in the terminal layer with a coordination number equivalent to that of bulk Mg. Given this structural similarity, the surface energy of the Mg-coated NbC particle can be reasonably approximated as that of pure Mg. This leads to *Δσ*_*cls*_ < 0, indicating spontaneous engulfment of NbC particles by the front during solidification of Mg(l)-Mg@NbC colloidal solution^31^. Furthermore, during solidification, Mg atoms at the advancing solidification front form Mg-Mg metallic bonds with the terminal Mg atoms on the surface of NbC particles, allowing the front to propagate through the Mg-coated NbC particles (Supplementary Fig. 12). This behaviour indicates a weak correlation between the orientation of NbC particles and the solidification front, resulting in the engulfment of randomly oriented particles. Experimentally, no apparent orientation relationships between the NbC particles and the Mg matrix were observed (Supplementary Figs. 2, 4, 6).

It is also notable that NbC possesses a significantly higher shear modulus (~230 GPa^32^) than in-situ formed precipitates^33–36^. These engulfed, chemically bonded high modulus NbC particles within the Mg matrix directly contribute to the effective strengthening^37^ of the Mg matrix.

In conclusion, this combined atomistic-level experimental and theoretical study reveals the formation of an ordered Mg layer bonded to NbC particles (Mg@NbC), which is responsible for the observed spontaneous wetting and engulfment. The resulting material exhibits high specific tensile strength and stiffness, surpassing many high-performance metals. To identify other metal-ceramic systems where the base molten metal spontaneously wets the ceramic nanoparticles, future design should specifically employ ab initio interface structure prediction and interfacial energy calculations. We believe DFT calculation can help in accelerating ceramic particle selection for metallic materials by investigating their interfacial interaction. Beyond magnesium, these insights provide a blueprint for designing next-generation structural and functional metal-ceramic systems and scalable nanocolloid-based processing.

## Methods

### Fabrication of Mg-NbC materials

NbC powder feedstock with three particulate sizes were used in this study, and their morphology and size distribution are given in Supplementary Fig. 14. The NbC particles with the average size (*D*_*50*_) of 906 nm were supplied from Companhia Brasileira de Metalurgia e Mineração (CBMM) coded as NbC_micron_. The NbC particles with the average size (*D*_*50*_) of 287 nm were acquired from American Elements coded as NbC_submicron_. The NbC particles with the average size (*D*_*50*_) of 15 nm were produced using plasma spray chemical synthesis^38–40^ coded as NbC_nano_ (Supplementary Figs. 15, 16). To produce NbC_nano_, a thermal plasma jet was first generated with a hybrid plasma spray system using Ar and H_2_ gases. Raw Nb metal powders with an average diameter of 45 µm (Kojundo Chemical Co. Ltd., Japan) were then introduced into the plasma to form high temperature Nb vapour through complete vaporisation. CH_4_ gas was also added to the plasma for carburisation of the Nb vapour to form NbC particles. The feed rate of the raw powders and the CH_4_ gas flow rate were adjusted to control the NbC nanoparticle size and Nb/C ratio of the NbC solid solution. For particle size distribution measurements, using ImageJ 1.53k software^41^, ~1500 particles were analysed for each batch. The NbC_nano_ and NbC_submicron_ powder feedstocks were cold pressed into 16 mm diameter pellets using a Specac Atlas 15 T manual hydraulic press with 0.5 ton pressure. In the case of the NbC_micron_ powder, 32 mm diameter pellets were produced by applying 1 ton pressure. The pressed NbC pellets were baked at 110 °C for 12 h and placed into a steel crucible containing molten Mg (99.95% purity) at 680 °C with a protective gas mixture of nitrogen and R134a gas in the ratio of 12:1 (nitrogen flow at 3 L/min, R134a at 0.25 L/min), under ambient pressure, to achieve pressure-less infiltration. During infiltration, the entrapped air in the NbC precursor pellet escapes in the form of bubbles to the molten metal surface. The infiltrated pellets were then solidified at 0.3 K/s cooling rate in protective gas. A pure Mg reference sample was also solidified at the same cooling rate. The volume fraction of the NbC particles in the Mg-NbC materials was calculated based on the measured weight difference between the cold pressed NbC pellet and as-solidified Mg-NbC materials, as well as the density of the pure Mg (1.78 g/cm^3^) and the NbC phase (7.8 g/cm^3^). The volume fraction of the NbC phase in as-solidified Mg-NbC_nano_, Mg-NbC_submicron_, and Mg-NbC_micron_ samples were 12.2 vol.%, 38.7 vol.%, and 53.8 vol.%, respectively. A summary of the infiltrated materials is listed in Supplementary Table 1. To demonstrate up-scalability for the preparation of a 1 kg batch Mg-NbC material, Mg-38.7 vol.%NbC_submicron_ infiltrated pellets were preheated at 200 °C for 2 h then added to a Mg melt at 680 °C under a protective atmosphere to obtain a final particle loading of 1 vol.%. After 15 min, the infiltrated Mg-NbC_submicron_ pellets were gently pressed into small fragments and manually stirred to release and disperse the NbC_submicron_ particles into the Mg melt. The resulting diluted Mg-1vol.%NbC_submicorn_ colloid was poured into a steel mould and solidified at a cooling rate of ~30 K/s.

### Mechanical characterisation

The micro-tensile specimens from as-solidified Mg-NbC_nano_ and Mg-NbC_submicron_ materials were prepared using a Thermo Fisher Scientific Scios 2 DualBeam focused ion beam-scanning electron microscope (FIB-SEM) and tested using a FemtoTools FT-NMT04 IN-SITU Nanoindentor under Field Emission-SEM (FE-SEM) observation in the Scios 2 instrument (Supplementary Fig. 17). The specimens were sectioned, lifted-out from the as-solidified Mg-NbC samples and then attached to a tungsten needle by Pt deposition into a prefabricated hole whose size conforms to the shape of the lifted sample. This was the fixed side in the tensile testing rig. The specimen was shaped into a dog-bone geometry with rectangular cross section (~10 μm gauge length and 12 μm^2^ cross sectional area). The dog-bone specimen was attached to a Si gripper that connected to a microforce sensing probe (FT-S200’000) within FT-NNMT04. The micro-tensile test was performed in the displacement control mode and a strain rate of 1 × 10^−4^ s^−1^. The video recording of the in-situ tensile tests is presented in supplementary files (Supplementary Videos 1–3).

The macro-tensile tests of the as-solidified Mg-1vol.% NbC_submicron_ and Mg-13vol.%NbC_micron_ materials and reference pure Mg were conducted on a Instron 5500 Universal Electromechanical Testing System with a constant cross head speed of 0.225 mm/min (2 × 10^−4^ s^−1^ initial strain rate) and the results are shown in Supplementary Fig. 7b and Supplementary Note Fig. 2c.

A Micro Materials NanoTest ALPHA with a Berkovich tip was used for nanoindentation tests with load-control to a maximum load of 100 mN with a 6.7 mN/s loading rate, held for 10 s, and unloaded at 6.7 mN/s. Hardness and elastic modulus are evaluated from the load-indentation depth curves.

### Strengthening contribution calculations

The two synergetic strengthening mechanisms in this work are particle-dislocation interaction (Orowan strengthening) and load-transfer strengthening. The Orowan strengthening^13^ can be expressed as:

3 $$\Delta {\sigma }_{{Orowan}}=\frac{\varphi {G}_{m}b}{{d}_{p}}{\left(\frac{6{V}_{P}}{\pi }\right)}^{1/3}$$

Where $\varphi$ is a coefficient with a value of 2. ${G}_{m}$ is the shear modulus of the matrix and is 17.3 GPa for Mg^42^. *b* is the Burgers vector of Mg and has value of 0.32 nm^43^. *V*_*p*_ denotes the volume fraction of NbC particles, and ${d}_{p}$ represents the NbC particle size. For each NbC catalogue, the *d*_peak-σ_, *d*_peak_, *d*_peak+σ_ particle sizes (Supplementary Table 1) were used to calculate the Orowan strengthening contribution.

The load-transfer strengthening increment is given by,

4 $$\varDelta {\sigma }_{{\mbox{LT}}}=\left(\frac{{E}_{p}}{{E}_{m}}-1\right){V}_{p}\,{\sigma }_{{YS}}^{m}$$

Where ${E}_{P}$ is the elastic modulus of NbC and is taken as 480 GPa^32^ and *E*_*m*_ is the elastic modulus of the Mg matrix and is taken as 45 GPa^42^. ${\sigma }_{{YS}}^{m}$ is the measured yield strength of Mg (35 MPa). ${V}_{p}$ is the volume fractions of the NbC particles. Further details of load-transfer contribution, $\varDelta {\sigma }_{{LT},}$ is given in Supplementary Note 2.

Grain boundary strengthening is considered for the polycrystalline Mg-NbC materials evaluated using macro-tensile test. The yield strength improvement arising from the Hall-Petch relationship^44,45^ can be written as $\Delta {\sigma }_{{GR}}=k\cdot {d}_{{Mg}-{NbC}}^{-1/2}-k\cdot {d}_{{Mg}}^{-1/2}$, where, *k* is the Hall-Petch coefficient^46^ with value of 150 MPa·μm^−1/2^, ${d}_{{Mg}-{NbC}}$ and ${d}_{{Mg}}$ are the average Mg grain size with and without NbC addition. The resulting $\Delta {\sigma }_{{GR}}$ is ~4.9 MPa for Mg-1 vol.%NbC_submicron_ material (${d}_{{Mg}-{NbC}}$ = 149 μm and ${d}_{{Mg}}$ = 417 μm), and ~2.6 MPa for Mg-13 vol.%NbC_micron_ material (${d}_{{Mg}-{NbC}}$ is 1.1 mm and ${d}_{{Mg}}$ is 5.0 mm).

### Microstructure observations

A Zeiss Crossbeam 340 SEM and Thermo Fisher Scientific Talos F200X were used to investigate the microstructure of the NbC powder feedstock and the Mg-NbC samples. Scanning transmission electron microscopy (STEM) images and energy dispersive x-ray spectroscopy (EDS) elemental maps were collected using the Talos F200X instrument.

Atomic-resolution STEM imaging and electron energy loss spectroscopy (EELS) mapping were conducted using an aberration corrected Nion UltraSTEM100 working at 100 kV. The microscope is equipped with a cold-FEG emitter with 0.3 eV native energy spread. With a fifth-order probe aberration corrector, the microscope optics can be configured to enable a probe size of <1 Å at 100 kV with a convergence semi-angle of 30 mrad and a probe current of 30 pA in the conditions used for these experiments. The semi-angular ranges for high-angle annular-dark-field (HAADF), medium-angle annular-dark-field (MAADF) images and annular-bright-field (ABF) images were 89 -195 mrad, 52–89 mrad (or 30–195 mrad when acquired simultaneously with ABF images), and 10–30 mrad, respectively. Electron energy loss spectra were acquired on a Gatan Enfina spectrometer that is retrofitted with a Quantum Detectors Merlin EELS hybrid pixel camera. EELS spectrum images (SIs) were acquired in “event-streamed” mode using a 36 mrad collection semi-angle. Leveraging the instrument’s high stability, characterised by negligible sample drift under experimental conditions, multiple consecutive SI scans with short pixel dwell times (3 or 5 ms/pixel) were accumulated and averaged. This approach enabled sufficient signal acquisition while minimising noise and sample damage through repeated acquisitions. Prior to data processing, the EELS SI dataset was denoised via the built-in principal component analysis (PCA) function in Gatan Microscopy Suite 3.6 (GMS 3.6), with careful inspection of residuals to ensure artefact-free reconstruction. Notably, the near-ideal Poisson noise characteristics of data collected using next generation hybrid pixel detectors make them particularly well-suited for PCA-based denoising, enabling enhanced signal extraction with minimal artefact introduction^47^. EELS elemental maps were generated in two ways. For SIs containing the Nb *M* edges (205 eV) and C *K* (284 eV) in close proximity, we used model-based EELS quantification function in GMS 3.6 to separate the two signal components for elemental mapping. This method was applied to create Fig. 1b, Supplementary Fig. 15d and Fig. 16d, and the C *K* map in Fig. 3c. For the Mg *K* (1305 eV, in Fig. 3c) and Nb *L*_*2,3*_ (2465 eV, in Fig. 3c, Supplementary Fig. 15e and Fig. 16e) edges, we first subtracted a decaying background preceding each edge using a fitted power-law function and then integrated the EELS edge intensity over a 50 eV window from each edge onset. EELS map intensities are shown using a false colour scale, where low intensity indicates lower relative elemental concentration, and higher contrast reflects a relative increase in concentration. We note that two consecutive SIs were acquired in the same region in Fig. 3c to cover a wide energy range involving all the aforementioned core-loss edges, while only C *K*, Nb *L*_*2,3*_ and Mg *K* were selected for illustration in the main text.

Cross-sections of the bulk samples were prepared using standard metallographic methods with the oil-based diamond suspensions. TEM specimens from the Mg-NbC samples were initially sliced from the bulk and ground to a thickness of ~100 µm, followed by dimpling using a Gatan dimple grinder system and subsequent thinning using ion milling with a Gatan PIPS 695 instrument that operated at 3–5 kV and beam angles of 3–5°. To remove damaged layers and any amorphous structure on the TEM foil surface, a final polishing step was performed at 1 kV with Ar^+^ ions at a 7° incident beam angle. Electron backscattered diffraction (EBSD) was performed to characterise the grain size of Mg in Mg-NbC samples. Specimens were placed in the Tescan Magna UHR-SEM tilted 70° from the horizontal plane towards the EBSD camera with a 30 kV electron beam, 13 nm spot size and 80–100 nm step size. The 4D-STEM based orientation/phase mapping was applied to produce crystallographic maps of the Mg-NbC_nano_ specimen at high resolution using a Talos F200X and NanoMegas ASTAR system at 200 kV, 2 nm step size. The data analysis was conducted by ATEX 5.0 software (Academic Edition)^48^.

The X-ray diffraction (XRD) spectrum for Mg-13vol.%NbC_micron_ sample was acquired using a Bruker D8 ADVANCE XRD diffractometer with a Cu K_α_ (*λ*= 0.1542 nm) radiation source, and the results are shown in Supplementary Note Fig. 2b. The candidate phases for matching are selected from Powder Diffraction File™ (PDF^®^) database.

### DFT calculations

The present ab initio molecular dynamics (AIMD) study employs the first-principles code VASP (Vienna Ab initio Simulation Package)^49^ that utilises the periodic boundary conditions (PBC). Supercells were built for modelling the interfaces between liquid Mg and NbC substrates. NbC {001} substrate has an equal number of Nb and C atoms at the surface, being electronically neutral and non-polar. The number of liquid Mg atoms for the systems is fixed to be 300, while the number of Nb atoms and C atoms are fixed to be 72. A tetragonal supercell was created with *a* = 3*a*_*0*_ for the Mg(l)/NbC_{001}_ interface, where *a*_*0*_ is the length of the axis of the conventional NbC cell with consideration of the thermal expansion at the simulation temperature^50^. The length of the *c*-axis is determined by the thickness of the NbC slab and the volume of the Mg atoms with the density at the simulation temperature^50^. The built supercells are summarised in Supplementary Table 4 and the related configurations are shown in Supplementary Fig. 18a. To avoid artificial interface interactions, a space of about 2.0 Å was made between the substrate atoms and the liquid Mg atoms.

VASP employs the electronic density-functional theory (DFT) within the projector-augmented wave framework^51^. The generalised gradient approximation (GGA-PBE) was used for the exchange and correlation terms^52^. The cut-off energy for the ab initio molecular dynamics simulations is 320.0 eV and Г-point in the Brillouin zone is used as there is a lack of symmetry in the liquid/solid interfaces^49,53^. The simulation temperature is set to be 1000 K.

A two-step approach was used: first AIMD simulations were performed with the substrate atoms fixed for about 300 steps (1.5 femtosecond (fs) per step). Then, the AIMD simulations are continued with relaxation of all the atoms. The simulations revealed thermal equilibration after about 1 picosecond (ps) (see Supplementary Fig. 18). This approach avoids risks of collective movements of atoms during the AIMD simulations^20^.

## Supplementary information

Supplementary Information

Description of Additional Supplementary Files

Supplementary Video 1

Supplementary Video 2

Supplementary Video 3

Transparent Peer Review file

## Data Availability

The source data that support the findings of this study are available in the figshare repository with the identifier (10.6084/m9.figshare.30060889^54^).
