# Supplementary material for: Ultrahigh strength magnesium via solidification of nanocolloid
Source: Nat Commun. 2026 Apr 10;17:5106. doi: 10.1038/s41467-026-71671-x (PMC13246850; doi:10.1038/s41467-026-71671-x)
Supplement: Supplementary file 1 — Supplementary Information [file 41467_2026_71671_MOESM1_ESM.pdf]

Ultrahigh strength magnesium *via* solidification of nanocolloid

Supplementary Information

Xinliang Yang[1], Hari Babu Nadendla[1*], Changming Fang[1], Shunsuke Nishi[2], Tomoki Matsuda[2], Makoto Kambara[2], Toshimi Tanaka[3], Masashi Dougakiuchi[2,4], Fengzai Tang[5], Geoffrey D. West[5], Shihao Wang[6,7*], Quentin M. Ramasse[6,7,8]

[1]BCAST, Brunel University of London, Kingston Lane, Uxbridge UB8 3PH, UK

[2]Division of Materials and Manufacturing Science, Graduate School of Engineering, The University of Osaka, 1-1 Yamadaoka, Suita, Osaka 565-0871, Japan

[3]Takeuchi Electric, 51-1, Hokuryo-cho, Matsue, 690-0816, Shimane, Japan

[4]Shimane Institute for Industrial Technology, 1 Hokuryo-cho, Matsue, 690-0816, Shimane, Japan

[5]WMG, University of Warwick, Coventry, CV4 7AL, UK

[6]SuperSTEM Laboratory, SciTech Daresbury Science and Innovation Campus, Keckwick Lane, Daresbury WA4 4AD, UK

[7]School of Chemical and Process Engineering, University of Leeds, Leeds LS2 9JT, UK

[8]School of Physics and Astronomy, University of Leeds, Leeds LS2 9JT, UK

*Corresponding authors: Hari Babu Nadendla, mtsthbn@brunel.ac.uk; Shihao Wang, swang@superstem.org

Contents

1. Supplementary Note 1 Assessment of colloid stability for larger particles
2. Supplementary Note 2 Assessment of load-transfer increment ($\Delta\sigma_{LT}$)
3. Supplementary Table 1 | The microstructural information of the infiltrated Mg-NbC materials.
4. Supplementary Table 2 | The properties of published metal matrix composites, which were used to generate the inset of Fig.2**b**.
5. Supplementary Table 3 | The properties of the published alloys and composites, which were used to plot Fig.2**d**.
6. Supplementary Table 4 | Inputs for the Mg(l)/NbC interfaces for the AIMD simulations.
7. Supplementary Figure. 1 | The microstructure of Mg-NbC_{nano} sample.
8. Supplementary Figure. 2 | The crystallographic analysis of Mg-NbC_{nano} specimen *via* 4D-STEM.
9. Supplementary Figure. 3 | The microstructure of Mg-$NbC_{submicron}$ sample.
10. Supplementary Figure. 4 | Distribution of $NbC_{submicron}$ particle in the Mg matrix.
11. Supplementary Figure. 5 | The microstructure of Mg-NbC_{micron} sample.
12. Supplementary Figure. 6 | Distribution of NbC_{micron} particle in the Mg matrix.
13. Supplementary Figure. 7 | Mg-1vol.%$NbC_{submicron}$ material prepared via dilution of the infiltrated Mg-$NbC_{submicron}$ material.
14. Supplementary Figure. 8 | SEM images of the micro-tensile samples.
15. Supplementary Figure. 9 | Specific stiffness versus specific hardness of the Mg-NbC materials and engineering alloys.
16. Supplementary Figure Fig. 10 | The interfacial region of ordered C_{NbC}-like Mg layer and follow-on hcp Mg.
17. Supplementary Figure Fig. 11 | Atomic arrangements of Mg(l)/$NbC_{\{001\}}$ interface.
18. Supplementary Figure Fig. 12 | The atomic density profile of Mg(l)/$NbC_{\{001\}}$ interface.
19. Supplementary Figure. 13 | Schematic atomic coordination of selected bond lengths for typical C atoms.
20. Supplementary Figure. 14 | NbC powder feedstock characterisation.
21. Supplementary Figure. 15 | NbC_{nano} observed *via* $[0\text{-}10]_{NbC}$ direction.
22. Supplementary Figure. 16 | NbC_{nano} observed *via* $[1\text{-}10]_{NbC}$ direction.

23. Supplementary Figure. 17 | The fabrication procedure of specimen for micro-tensile testing.
24. Supplementary Figure. 18 | Configuration of *ab initio* molecular dynamics (AIMD) simulation.

Supplementary Note 1: Assessment of colloid stability for larger particles

The influence of gravity must be considered when assessing colloidal stability, particularly for larger particles. Stoke's equation[s1] is used to evaluate the sedimentation behaviour of the molten Mg-NbC system. Calculated sedimentation time shown in Supplementary Note Fig. 1**a** indicates that a 0.1 vol.% suspension of micron-sized NbC particles requires approximately 10 hours to sediment through a 100 mm depth in the crucible. When the particle diameter decreases to 100 nm, the sedimentation time increases significantly to around 1000 hours, which exceeds the duration typical of standard foundry casting processes.

To justify the colloidal stability, an Mg-13vol.% NbC_{micron} colloidal solution was prepared by incorporating micron-sized NbC particles into molten Mg, followed by mechanical mixing using an impeller at 200 rpm. Then the suspension was held stationary for one hour before being allowed to solidify in the furnace under a slow, controlled cooling rate (see Supplementary Note Fig. 2). A local hardness measurement approach was used to assess the macroscopic uniformity of particle distribution. Supplementary Note Fig. 1**b** shows the spatial variation in Vickers hardness along the vertical cross-section of an ingot solidified from a Mg-13vol.% NbC_{micron} colloid. If sedimentation occurs, a sudden change in hardness would be observed. As seen in Supplementary Note Fig. 1**b**, the measured Vickers hardness remained consistent along the vertical section of the solidified ingot, indicating that no noticeable sedimentation occurred. This demonstrates that, despite the relatively large particle size, a stable Mg(l)-NbC colloid can be achieved.

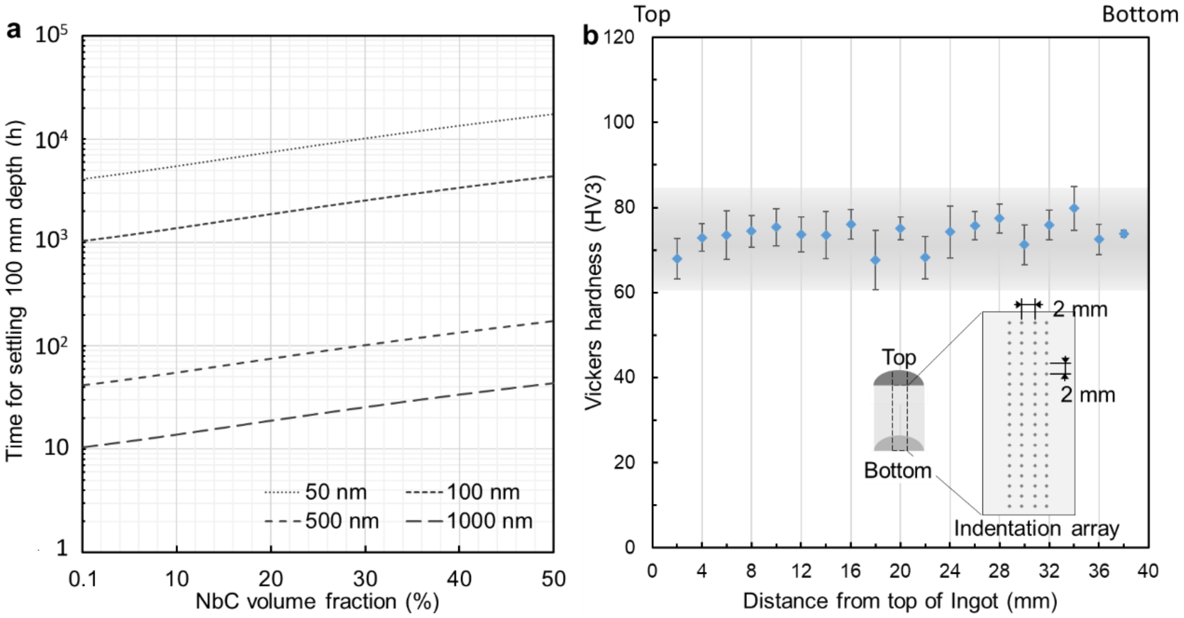

Supplementary Note Figure. 1 | The colloid stability assessment. **a,** The calculated settling time of NbC particles vs volume fraction. **b,** The Vickers hardness as a function of the position in the sample solidified from Mg-13vol.%NbC_{micron} colloid. The unform hardness of the sample from top to the bottom of the as-solidified ingot suggesting stability of colloid without sedimentation of NbC particles at the bottom of the crucible.

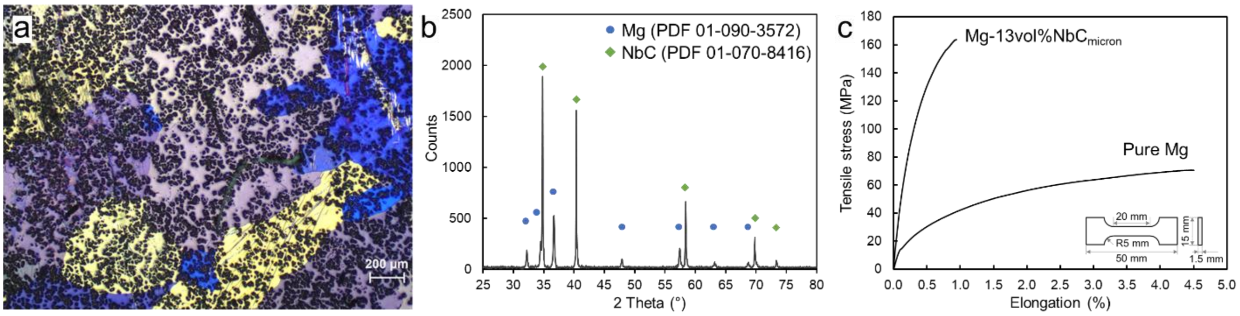

Supplementary Note Figure. 2 | The as-solidified sample from Mg-13vol.%NbC_{micron} colloid. **a,** Colour contrast represents the individual Mg grains with different orientation polarised optical microstructure. The dark spots in the Mg grains represent the NbC_{micron} dispersions. **b,** XRD spectrum showing the phase constitution in this sample. **c**. Tensile stress-strain curves of the Mg-13vol.%NbC_{micron} sample and pure Mg sample under same solidification conditions.

Supplementary Note 2: Assessment of load-transfer increment ($\Delta\sigma_{LT}$)

The load-transfer contribution to composite strengthening is governed by (i) the stress required by elastic compatibility between the matrix and the particles, and (ii) the maximum stress that can be transmitted across the particle-matrix interface without interfacial debonding. Accordingly, the effective load-transfer increment[s2, s3] is defined as:

$$\Delta\sigma_{\mathrm{LT}}(V_p, d) = \min\left[\Delta\sigma_{\mathrm{LT,Voigt}},\ \Delta\sigma_{\mathrm{LT,max}}(V_p, d)\right] \qquad \text{SEq. (1)}$$

Here, $\Delta\sigma_{\mathrm{LT,Voigt}}$represents the load-transfer requirement imposed by elastic strain compatibility at the onset of matrix yielding while $\Delta\sigma_{\mathrm{LT,max}}$represents the upper bound on load transfer imposed by interfacial strength, V_p is the particle volume fraction and d is particle size. This formulation ensures that the predicted load transfer does not exceed the physical stress-transfer capacity of the particle-matrix interface. The use of the minimum operator reflects the fact that both constraints must be satisfied simultaneously. If $\Delta\sigma_{\mathrm{LT,Voigt}} < \Delta\sigma_{\mathrm{LT,max}}$, the interface is sufficiently strong to transmit the required stress and load transfer is governed by elastic modulus partitioning (perfect load-transfer regime). Conversely, $\Delta\sigma_{\mathrm{LT,Voigt}} > \Delta\sigma_{\mathrm{LT,max}}$, the interface cannot sustain the required stress, interfacial debonding occurs, and load transfer becomes interface limited. Thus, the effective load-transfer increment is always the smaller of what elastic compatibility demands and what the interface can physically sustain.

Voigt load-transfer requirement, $\Delta\sigma_{\mathrm{LT,Voigt}}$: Under the iso-strain (Voigt) condition, the Mg matrix and NbC particles experience the same axial strain during loading. When the matrix reaches its yield stress σ_y^m, the stiffer NbC particles must carry a proportionally higher stress determined by the elastic modulus contrast between the phases. This requirement leads to the classical expression[s4, s5]: $\Delta\sigma_{\mathrm{LT,Voigt}} = (\frac{E_p}{E_m} - 1)V_p\ \sigma_y^m$. Importantly, this term is independent of particle size and quantifies the additional stress that must be transferred to the particles in order, for the composite, to satisfy elastic strain compatibility at the onset of matrix yielding.

Interface-limited maximum transferable stress, $\Delta\sigma_{\mathrm{LT,max}}(V_p, d)$: Even if elastic compatibility demands a certain level of load transfer, that load can only be realised if the particle-matrix interface can transmit the required stress without debonding. For

spherical particles, the particle-matrix interfacial area per unit composite volume scales[s3] as: $A_v = \frac{6V_p}{d}$ such that decreasing particle size increases the available interfacial area. However, the stress required to initiate interfacial debonding is governed by fracture energy, rather than by interfacial area alone. For a stiff spherical inclusion embedded in a more compliant matrix, the stress required to initiate interfacial debonding[s6] is given by

$$\sigma_{\text{debond}}(d) = \frac{1}{3(1-\nu_m)}\sqrt{\frac{16\,\Gamma\,E_m(1+\nu_m)}{d}} \qquad \text{SEq. (2)}$$

where Γ is the interfacial fracture energy, and ν_m is the Poisson's ratio of the matrix. Using $\Gamma = 1.54$ J·m^{-2} from DFT calculations and Mg elastic constants[53] ($E_m = 45$ GPa, $\nu_m = 0.35$), the debonding stresses for 15 nm, 287 nm, and 906 nm particles are calculated to be approximately 5.12 GPa, 1.17 GPa, and 0.66 GPa, respectively. The conservative estimate of maximum load-transfer contribution that can be sustained without debonding is therefore written as: $\Delta\sigma_{\text{LT,max}}(V_p, d) = \eta\, V_p\, \sigma_{\text{debond}}(d)$ where η is stress-transfer efficiency factor (commonly $\eta \approx 0.5$ for particulate composites), introduced to account for non-uniform interfacial stress distributions and to avoid overestimating the load-transfer capacity of the interface. Smaller particles require higher stresses to debond and therefore permit higher interface-limited load transfer. These interfacial stresses substantially exceed the load-transfer requirement imposed by matrix yielding ($\sigma_y^m = 35$ MPa). Consequently $\sigma_{\text{LT,Voigt}} < \sigma_{\text{LT,max}}(V_p, d)$ for all three particle catalogue studied here and the effective load-transfer increment[s2, s4] expression (Supplementary Equation 1) reduces to $\Delta\sigma_{\text{LT}}(V_p, d) = \Delta\sigma_{\text{LT,Voigt}}$. This confirms that the Mg - NbC composites operate in the perfect load-transfer (Voigt-limited) regime, with particle size influencing the robustness of load transfer but not limiting its magnitude within the investigated range.

Supplementary Table 1 | The microstructural information of the infiltrated Mg-NbC materials.

	Mg average grain size (μm)*	NbC size (volumetric) (nm) (D_{10}, D_{50}, D_{90})	NbC size (nm) ($d_{peak-\sigma}$, d_{peak}, $d_{peak+\sigma}$ values from lognormal distribution)	NbC volume fraction (%)
Mg-NbC_{nano}	271	9, 15, 23	7, 9, 14	12.2
Mg-$NbC_{submicron}$	243	142, 287, 518	40, 49, 164	38.7
Mg-NbC_{micron}	530	500, 906, 1771	222, 281, 730	53.8

*Mg grain size (equivalent circular diameter) was extracted from the EBSD data.

Supplementary Table 2 | The properties of the published metal matrix composites, which were used to plot Fig.2b inset.

	composition	Volume fraction (V_p) (%)	Reinforcement dimension (nm)	Yield strength contribution (Δ_{YS}) (MPa)
This work	Mg-$NbC_{submicron}$	38.7	287	549
	Mg-NbC_{nano}	12.2	15	401
Mg MMCs	Mg/4wt.%CNTs[s7]	5.3	40	30
	Mg-1Cu/0.54wt.%GNPs[s8]	0.5	15	122
	Mg/0.3wt.%Ni@CNT[s9]	0.4	20	80
	AZ31/1.0wt.%CNTs[s10]	1.3	30	35
	AZ31/21vol.%Ti_p[s11]	21	2700	95
	AZ31/3wt.%GNP[s12]	2.4	20	12
	AZ91D/20vol.%Ti_2AlC[s13]	20	10000	62
	AZ31/2wt.%Al_2O_3[s14]	0.9	50	35
	AZ91/20vol.%SiC_p[s15]	20	10000	95
	AZ91D/1wt.%CNT[s16]	1.3	200	63
	AZ31B/0.95vol.%CNT[s17]	0.95	15	76
	Mg/0.97vol.%Al_p+0.66vol.%Al_2O_3[s18]	1.6	50	89
	ZK60/0.1wt.%GO[s19]	0.1	3.9	31
	AZ91/5.0wt.%Zn@Ti_p[s20]	5	15000	24
Al MMCs	Al/5vol.%MgO@GL[9]	5	5	400
	Al/5wt.%CuO[s21]	2.2	20	207
	Al-6Cu-0.4Mn/20vol.%SiC_p[s22]	20	3000	88
	Al6061/15wt.%SiC[s23]	13	500	81
	Al-10Si-1Mg/5vol.%TiB_2[s24]	5	50	40
Ti MMCs	Ti-6Al-4V/1wt.%TiB_2[s25]	1.6	44	189
	Ti-6Al-4V/5vol.%(TiB+TiC)[s26]	5	200	330
	Ti-6Al-4V/5vol.%TiB[s27]	5	150	305
	Ti-6Al-4V/2wt.%GNPs[s28]	3.9	20	145
	Ti-6Al-4V/0.5wt.%GNPs[s29]	1.7	10	260
	Ti/0.8vol.%BNNSs[s30]	0.8	100	170
	Ti/0.5wt.%SiCNWs[s31]	0.7	200	206
	Ti-6Al-4V/0.15wt.%Graphene[s32]	0.5	130	128
	Ti-6Al-4V/0.2wt.%GNPs[s33]	0.7	5	186
	Ti-6Al-4V/0.5wt.%GNS[s34]	1.7	100	361
	Ti-6Al-4V/5.1vol.%TiB[s35]	5.1	130	201
	Ti-6Al-4V/4vol.%Ti_5Si_3+3.4vol.%TiB_w[s36]	7.4	500	320
	Ti-22Al-27Nb/TiB[s37]	6.5	1000	367
Fe MMCs	15-5PH/2wt.%WC[s38]	1.1	2000	371
	Fe-18Cr-11Ni-2Mn-2Mo-1Si/1.5vol.%ZrO_2[s39]	1.5	44	192
	17-4PH/2wt.%TiN_{nano}[s40]	2.9	90	139

The presented properties of the metal matrix composite (MMC) materials were extracted from the referenced literature.

Supplementary Table 3 | The properties of the published alloys and composites, which were used to plot Fig.2d.

	composition (wt.%)	Processing method	Tensile yield strength (MPa)	Ultimate tensile strength (MPa)	Elongation (%)	Elastic modulus (GPa)*	Density (g/cm^3)
This work	Mg-NbC$_{submicron}$	casting	584	678	10.1	130	4.14
	Mg-NbC$_{nano}$	casting	436	526	11.9	82	2.53
Al	AlMnMgSc[55]	AM L-PBF	632	576	7.1	75	2.77
Ti	TiAl6V4[56]	Hot rolled	1660	1649	2.3	114	4.43
Steel	F82H[57]	/	519	457	10.2	220	7.89
	Fe-0.01C[58]	High-pressure torsion	1650	1130	2.8	210	7.87
	304 SS[59]	/	710	505	43	193	7.93
	IF steel[60]	Heat treated	213	125	5.2	210	7.87
Ni	RR1000[61]	Powder metallurgy	699	619	14.0	200	8.50
W	W-5Re[62]	Hot & cold rolled	1146	1299	3.2	411	19.3
Al MMCs	AlSi10Mg/TiB$_2$[63]	AM L-PBF	550	470	18	82	2.80
	Al-0.6RGO[64]	Powder metallurgy	336	264	10.7	75	2.70
Ni MMC	Ni-3wt.%SiC[65]	Spark plasma sintering	702	640	7.04	203	8.91

The presented properties of the advanced materials were extracted from the referenced literature. *The elastic modulus of the Mg-NbC samples were measured using the load-displacement curves during unloading period of the nanoindentations[s41].

Supplementary Table 4 | Inputs for the Mg(l)/NbC interfaces for the AIMD simulations.

System	Shape of cell	Lattice parameters (Å)	Number of atoms	Surface polarity
Mg(l)/NbC$_{\{001\}}$	Tetragonal	a =13.52<br>c = 52.29	Mg: 300<br>Nb: 72<br>C: 72	Non-polar

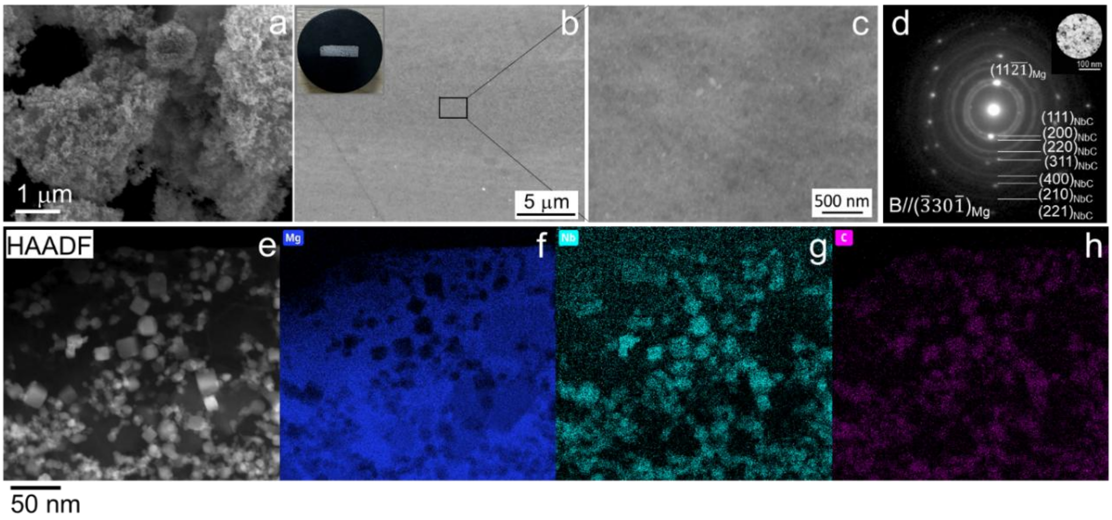

Supplementary Figure. 1 | The microstructure of Mg-NbC$_{nano}$ sample. **a,** SEM image of NbC$_{nano}$ particulates feedstock. **b,** SEM image of the as-solidified Mg-NbC$_{nano}$ sample showing uniform structure. The inset in **b** is cross-sectional image of pressure-less infiltrated and solidified bulk Mg-NbC$_{nano}$ sample mounted in Bakelite for metallography investigations. **c,** High magnification image of the region shown in **b** further confirming uniform structure. **d,** A selected area diffraction pattern from a Mg-NbC$_{nano}$ sample collected using TEM revealing the diffraction ring for the NbC crystal structure together with the Mg pattern, suggesting the presence of a high number density of randomly oriented NbC nanoparticles within a Mg grain. The inset is the bright field TEM image showing the area (~200 nm diameter) from which the diffraction pattern was taken. **e,** HAADF-STEM image of the same sample, and corresponding STEM/EDS elemental maps of **f,** Mg; **g,** Nb; and **h,** C elements.

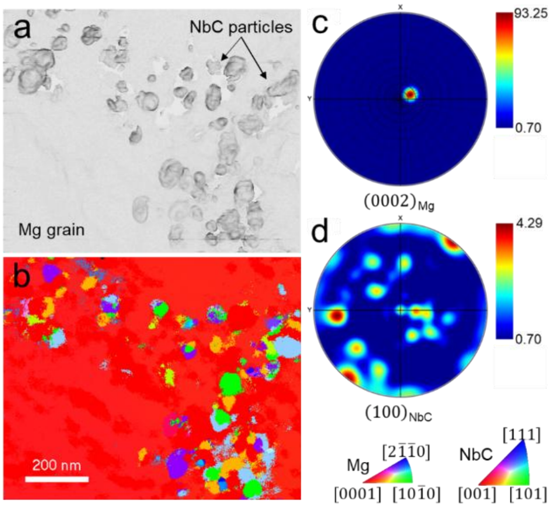

Supplementary Figure. 2 | The crystallographic analysis of Mg-NbC$_{nano}$ specimen *via* 4D-STEM showing the randomly oriented NbC in one Mg grain. **a,** Correlation coefficient map. **b,** inverse pole figure map. **c,** Mg pole figure in (0002)$_{Mg}$ pole. **d,** NbC pole figure in (100)$_{NbC}$ pole.

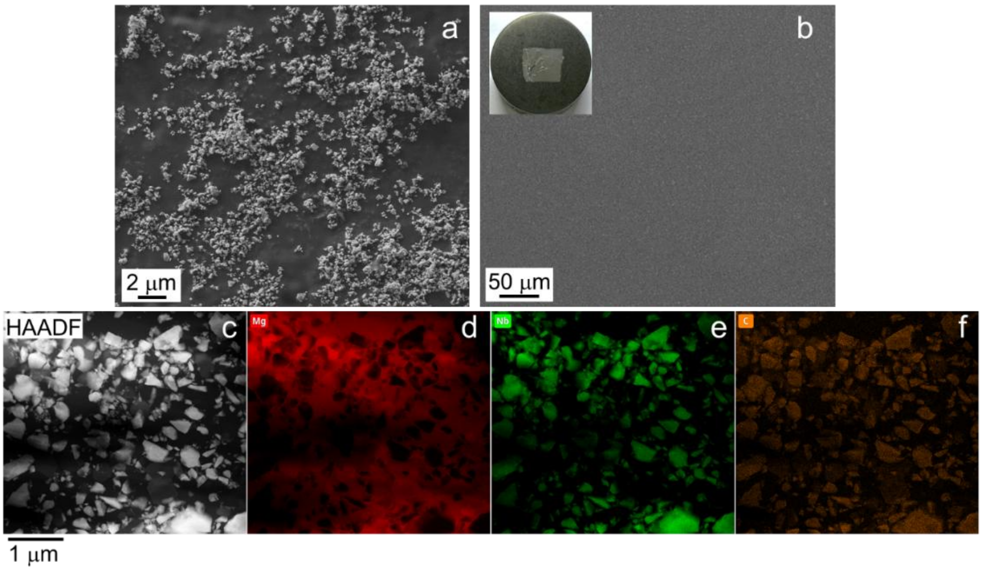

Supplementary Figure. 3 | The microstructure of Mg-NbC$_{submicron}$ sample. **a,** SEM image of NbC$_{submicron}$ feedstock, **b,** SEM image of the as-solidified sample and the inset is a photograph of the bulk sample. **c,** HAADF-STEM image and corresponding STEM/EDS elemental maps of **d,** Mg; **e,** Nb; and **f,** C elements.

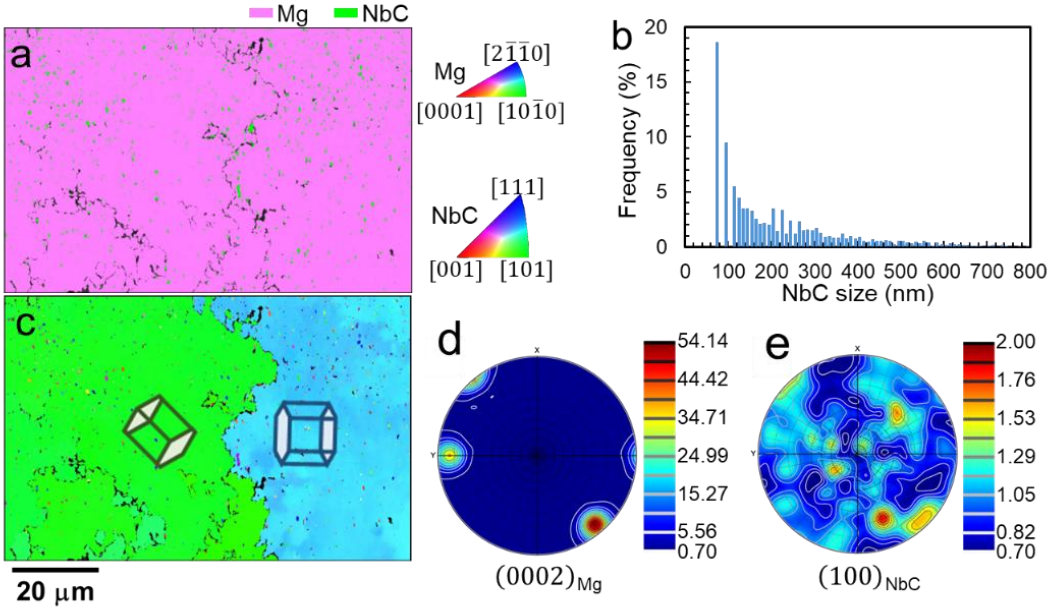

Supplementary Figure. 4 | Distribution of NbC$_{submicron}$ particle in the Mg matrix. **a,** Phase map of the Mg-NbC$_{submicron}$ specimen. **b**, Particle size distribution of the indexed NbC. The NbC particles with size lower than the indexing step size (80 nm) are not identified using the EBSD method. **c**, Inverse pole figure map of the same region as in **a**. **d,** (0002)$_{Mg}$ pole figure, and **e,** (100)$_{NbC}$ pole figure. This investigation shows a large number of randomly oriented submicron NbC particles that are dispersed within Mg grains. The absence of particle segregation at grain boundaries indicates that the solidifying Mg front did not push sub-micron particles during solidification.

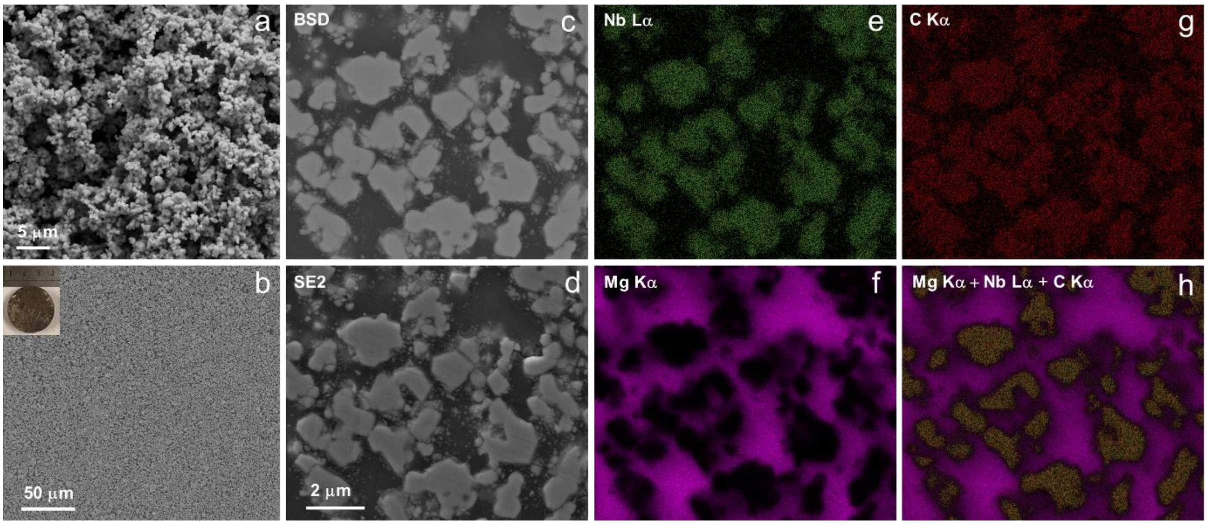

Supplementary Figure. 5 | The microstructure of Mg-NbC_{micron} sample. **a,** SEM image of NbC_{micron} particulates feedstock. **b,** SEM image of the as-solidified Mg-NbC_{micron} sample showing uniform structure. The inset in **b** is a photo of the pressure-less infiltrated and solidified bulk Mg-NbC_{micron} sample with a 32 mm diameter. **c,** Higher magnification image of the sample shown in **b** showing micron scale NbC particle distribution in Mg matrix. **d-h,** Secondary electron image and the corresponding SEM/EDS elemental maps of **e,** Nb; **f,** Mg; **g,** C; and **h,** combined map for Mg + Nb + C elements.

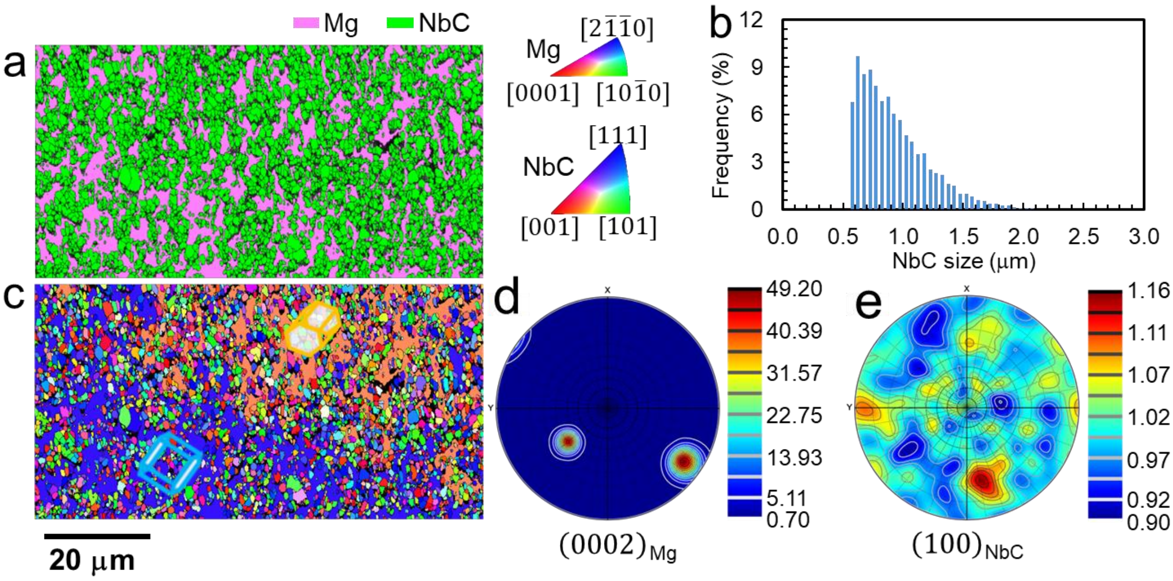

Supplementary Figure. 6 | Distribution of NbC_{micron} particle in the Mg matrix. **a,** Phase map of the Mg-NbC_{micron} specimen. **b**, Particle size distribution of the indexed NbC particles. **c**, Inverse pole figure map of the same region as in **a**. **d**,$(0002)_{Mg}$ pole figure and **e**, $(100)_{NbC}$ pole figure. This investigation shows a large number of randomly oriented NbC_{micron} particles dispersed within Mg grains.

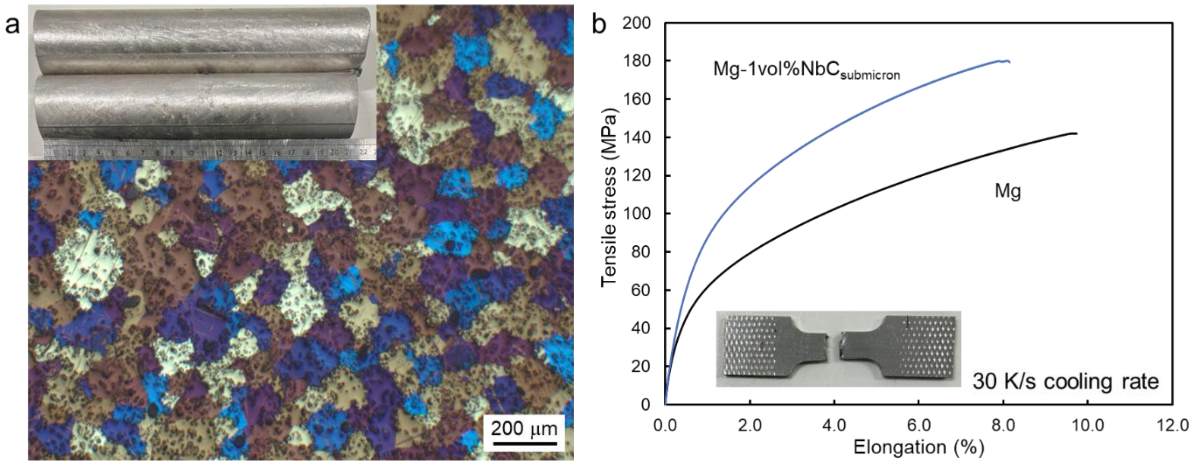

Supplementary Figure. 7 | Mg-1vol.%NbC$_{submicron}$ material prepared via dilution of an infiltrated Mg-38.7 vol.%NbC$_{submicron}$ pellet. **a,** Microstructure of the Mg-1vol.%NbC$_{submicron}$ material, the inset shows a photograph of the as-solidified ingots. **b**, Tensile stress-strain curves of the Mg-1vol.%NbC$_{submicron}$ material and pure Mg, both solidified at a cooling rate of 30 K/s. The inset shows a photograph of the macro-tensile tested Mg-1vol.%NbC$_{submicron}$ specimen.

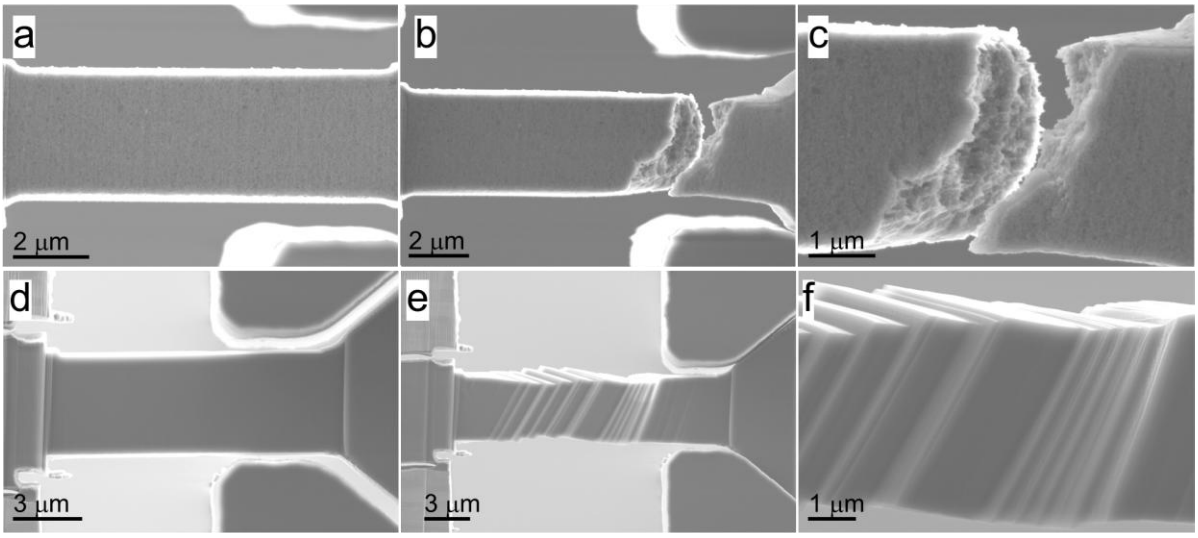

Supplementary Figure. 8 | SEM images of the micro-tensile samples. a, b, Mg-NbC$_{nano}$ specimen before and after tensile deformation. **c,** The enlarged image of the fracture surface shown in **b**. **d, e,** Pure Mg specimen before and after tensile deformation. **f**, The enlarged image of the basal slip activated area shown in **e**.

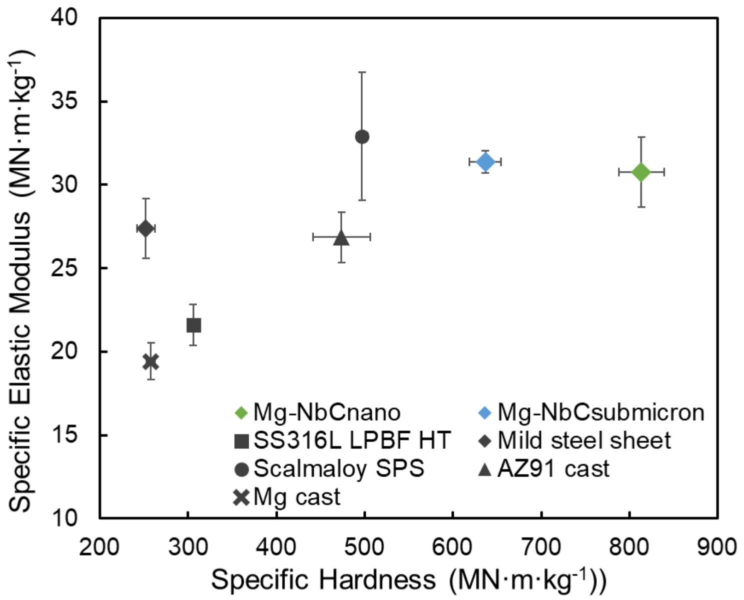

Supplementary Figure. 9 | Specific stiffness versus specific hardness of the Mg-NbC and engineering alloys. The nanoindentation test results of the Mg-NbC_{nano} and Mg-$NbC_{submicron}$ show an advanced combination of specific stiffness and hardness compared to the benchmark engineering alloys. Error bars represent the standard deviation of the measured values in the nanoindentation testing.

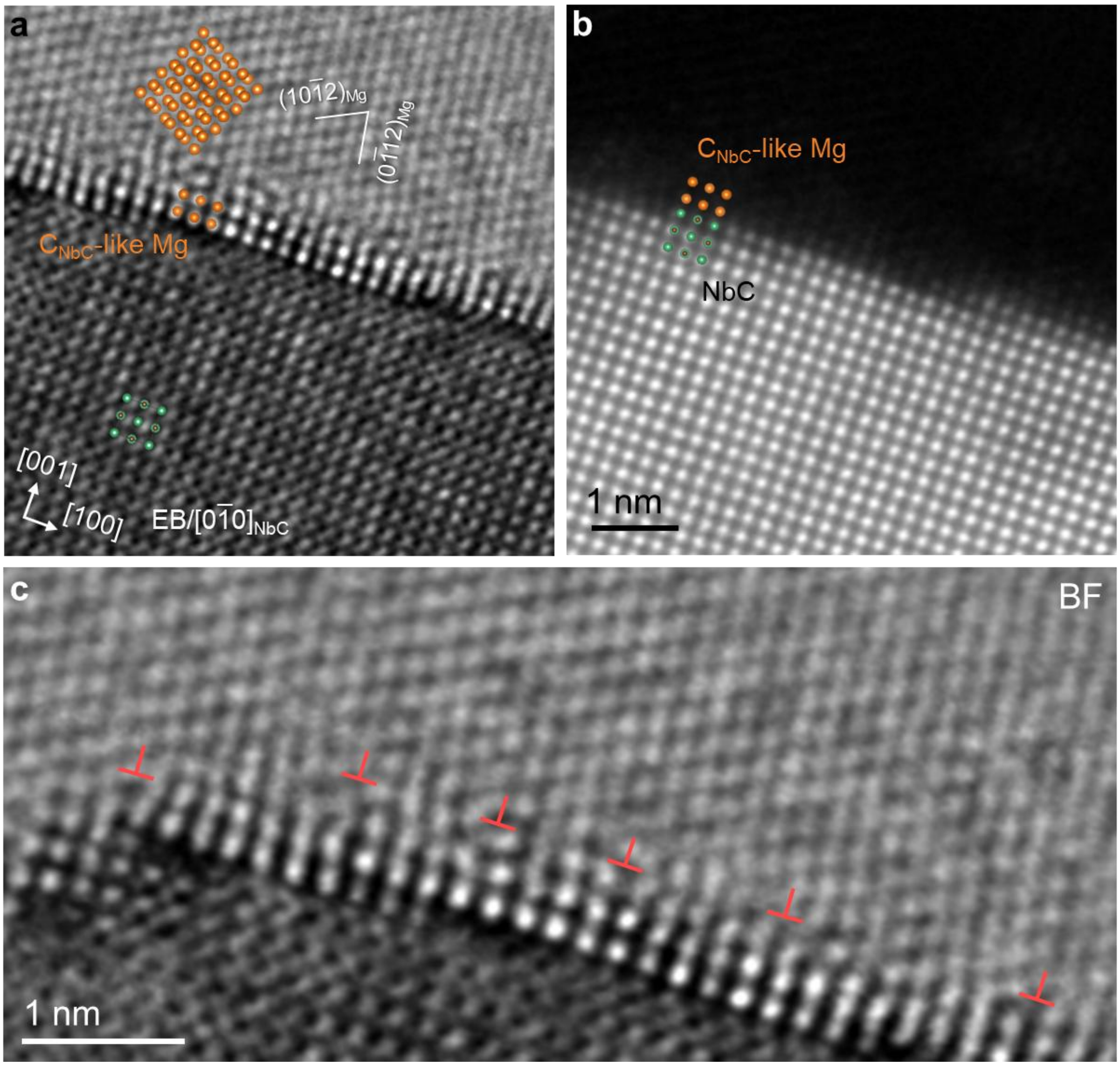

Supplementary Figure. 10 | The interfacial region of ordered C_{NbC}-like Mg layer and follow-on HCP Mg crystal structure. **a.** BF STEM image of the interfacial area revealing the transitioning of atomic arrangements from the C_{NbC}-like Mg terminating layer to the hexagonal close packed Mg crystal structure. **b,** HAADF STEM image showing the interfacial area of NbC {001} surface and the C_{NbC}-like Mg terminating ordered layer. **c**, the enlarged area in **a** exhibiting the microstructural transitioning across the interface, at which misfit dislocations are identified The incident beam is parallel to the zone axis $[0\text{-}10]_{NbC}$, and close to the $[-2201]_{Mg}$. Due to the presence of steps on the NbC surface and the ordering behaviour of Mg, the scattered electron signals from the Mg terminal layer can overlap with that from NbC step when projected along either the <100> or <110> directions along the NbC {001} termination plane. This overlap results in the image displaying an apparent interfacial multilayer.

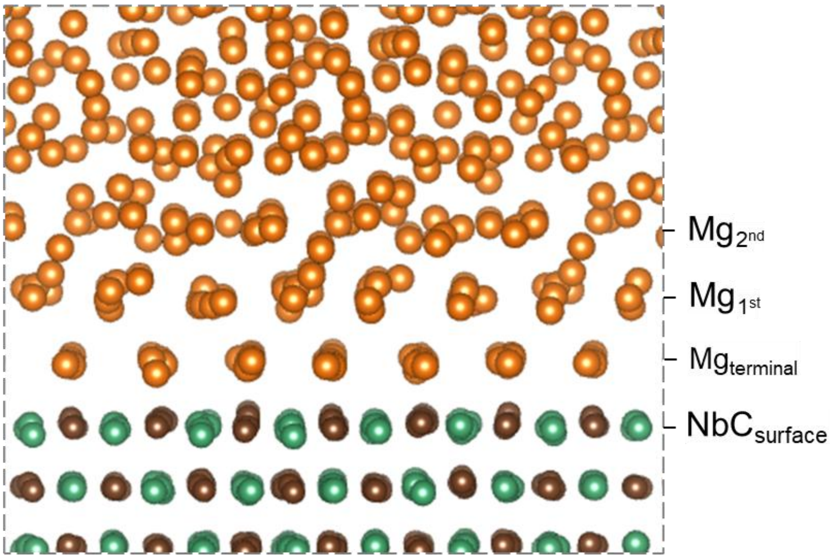

Supplementary Figure. 11 | Atomic arrangements of Mg(l)/NbC$_{\{001\}}$ interface. A snapshot of the atomic arrangements of Mg(l)/NbC{001} interface via <110>$_{NbC}$ direction equilibrated at 1000K. Green, brown and orange spheres represent Nb, C and Mg atoms, respectively.

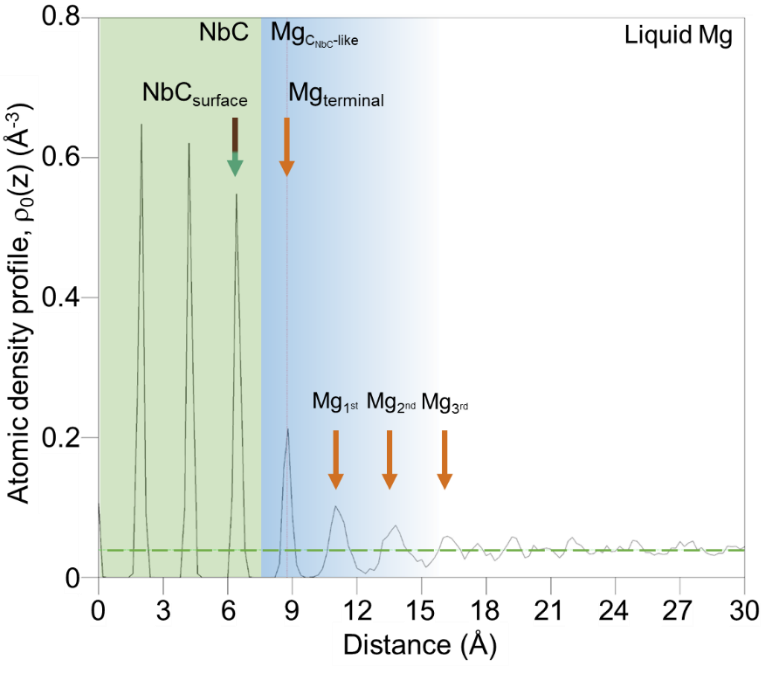

Supplementary Figure. 12 | The atomic density profile of Mg(l)/NbC$_{\{001\}}$ interface. There are three recognisable peaks. The terminating Mg atoms form one almost isolated symmetrical peak, marked by the red dotted line. The 1st peak is also symmetrical with reduced height. There is a finite atomic density between the 1st and the 2nd Mg layer. The 2nd peak is broad, consisting of a shoulder at 13.2 Å and a peak at 13.8 Å, indicating different stacking and bonding. There is a high density between the 2nd and 3rd Mg layer. The atomic density of 3rd Mg peak is close to the average value of liquid Mg (dashed green line). The atoms beyond 3rd peak behaves liquid like.

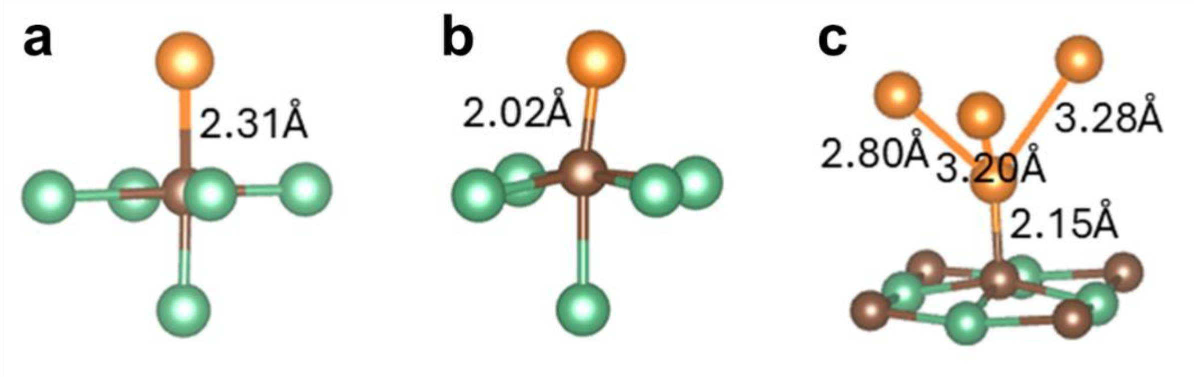

Supplementary Figure. 13 | Schematic atomic coordination of selected bond lengths for typical C atoms (the dark brown spheres). a, b, one Mg (the orange spheres) bonded to C atom at the Mg(l)/$NbC_{\{001\}}$ interfaces. The green spheres represent Nb atoms. **c**, schematic structure of atomic coordination of one terminating Mg atom in between outmost $NbC_{\{001\}}$ surface and 1st Mg layer.

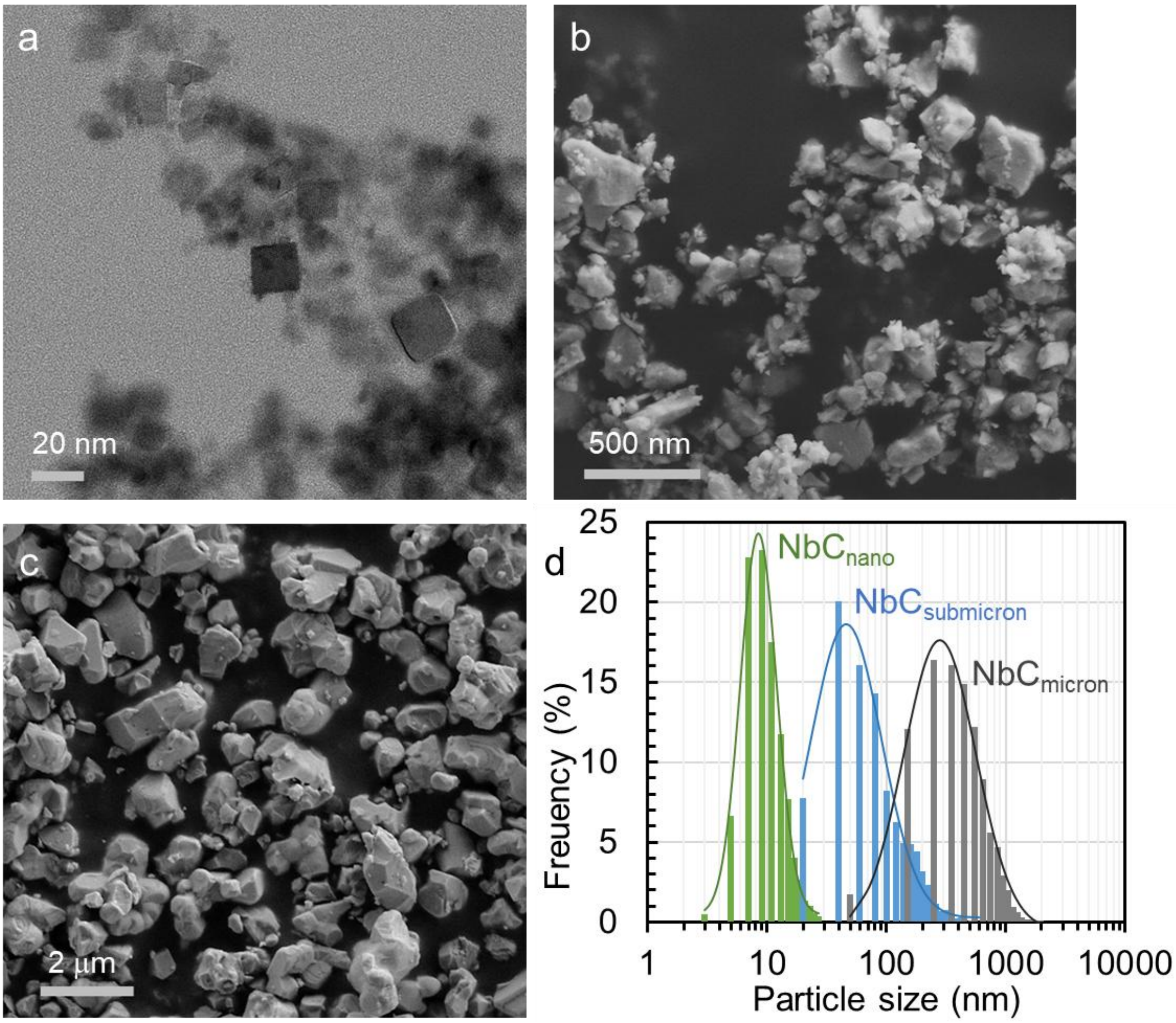

Supplementary Figure. 14 | NbC powder feedstock characterisation. **a,** TEM image of NbC_{nano}; **b,** SEM image of $NbC_{submicron}$; **c,** SEM image of NbC_{micron}, and **d,** their particle size

distribution, the solid curves are the fits to lognormal distribution and the computed $d_{peak-\sigma}$, d_{peak}, $d_{peak+\sigma}$ particle sizes are tabulated in Supplementary Table 1.

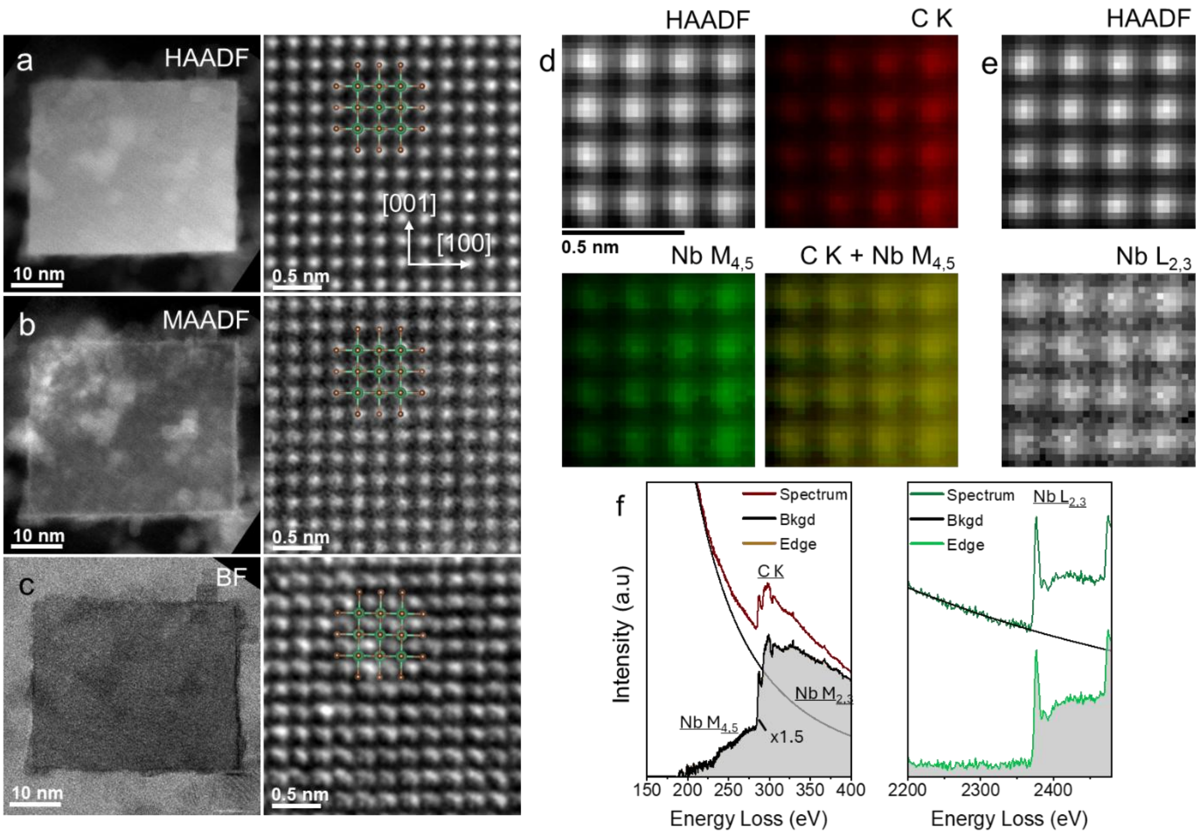

Supplementary Figure. 15 | NbC$_{nano}$ observed *via* the [0-10]$_{NbC}$ direction. a, HAADF STEM, **b,** MAADF STEM, **c,** BF TEM images at low and high magnification. **d,** HAADF image and corresponding EELS elemental maps of Nb $M_{4,5}$ edges, C K edge, and combined Nb + C map. **e,** HAADF image and corresponding STEM map of Nb element using Nb $L_{2,3}$ edges. **f,** EELS spectrums corresponding to **d** and **e**. The insets to (a-c) illustrate the atomic configuration of the NbC crystal**,** where green, dark brown and orange spheres represent Nb, C and Mg atoms, respectively.

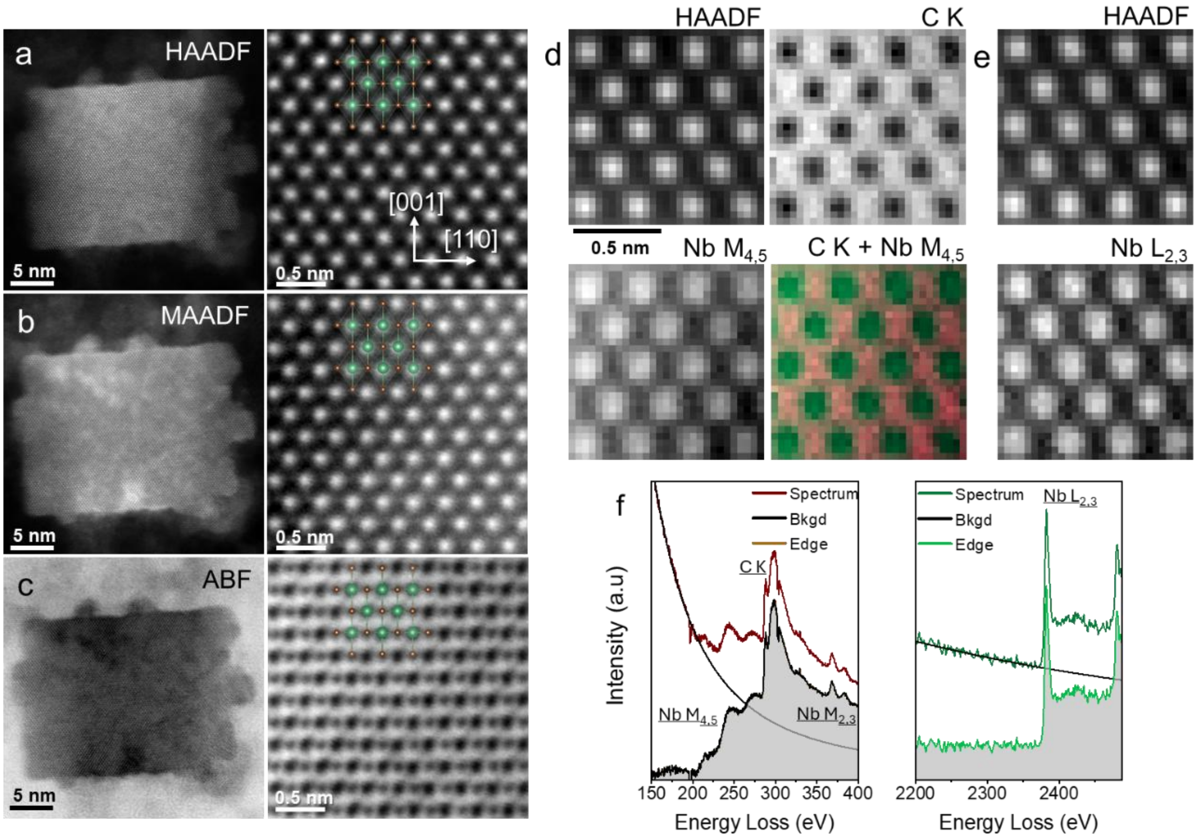

Supplementary Figure. 16 | NbC_{nano} observed *via* the $[1\text{-}10]_{NbC}$ direction. a, HAADF STEM, **b,** MAADF STEM, and **c,** ABF STEM images at low and high magnification. **d,** HAADF image and corresponding EELS elemental maps of Nb $M_{4,5}$ edges, C K edge, and combined Nb + C map. **e,** HAADF image and corresponding STEM map of Nb element using Nb $L_{2,3}$ edges. **f,** EELS spectra corresponding to **d** and **e**. The insets to (a-c) illustrate the atomic configuration of NbC crystal**,** where green, dark brown and orange spheres represent Nb, C and Mg atoms, respectively.

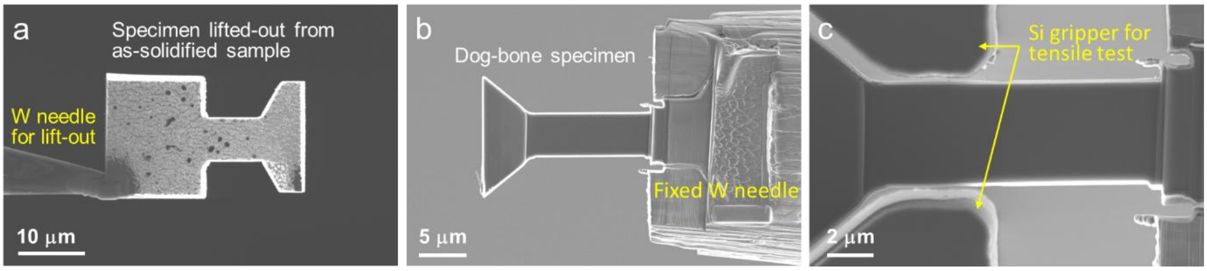

Supplementary Figure. 17 | The fabrication procedure of specimen for micro-tensile testing. **a,** Lift-out of the specimen from as-solidified sample. **b**, Attachment of the lift-out specimen to a preliminarily fabricated tungsten needle. **c**, Attachment of the silicon gripper for micro-tensile testing of the specimen.

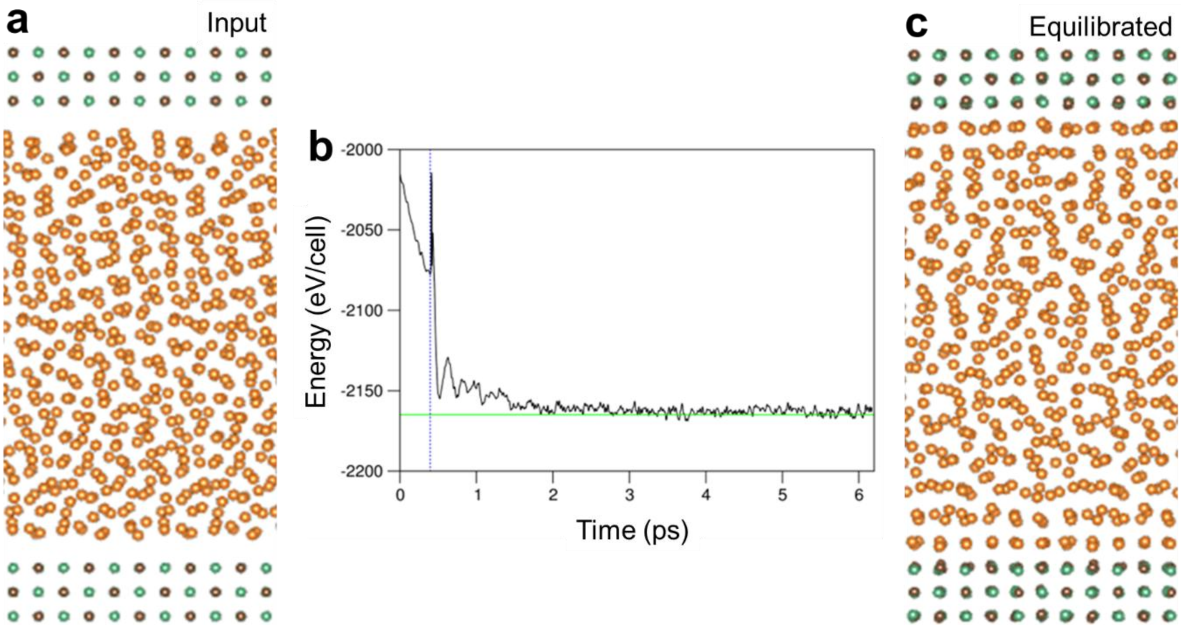

Supplementary Figure. 18 | Configuration of *ab initio* molecular dynamics (AIMD) simulation. a, Atomic arrangements for the input Mg(l)/$NbC_{\{001\}}$ interfaces. **b,** Dependence of the total valence electron energy on simulation time. **c,** Snapshots for the equilibrated Mg(l)/$NbC_{\{001\}}$ interface. Input configuration with setting of a 2.0Å distance between the substrate atoms and the liquid Mg atoms leads the system in high energy state. A two-step approach was used: first AIMD simulations were performed with the substrate atoms fixed for about 300 steps (1.5 femtosecond (fs) per step). Then, the AIMD simulations are continued with relaxation of all the atoms. Once simulations start, the liquid Mg atoms were observed to be moving around. The liquid atoms near the NbC substrate were observed to move towards the fixed substrate atoms, gradually forming chemical bonding with the substrate atoms, forming an Mg coated NbC (Mg@NbC) surface. The energy decreases quickly with time. In the 2nd step, when the substrate atoms are released, the energy rises sharply. Then it decreases quickly and then, gradually approaches the equilibrium value within another 2ps. Later, the total valence-electron energy of the system varies around the equilibrium value. When we equilibrated the system for another 4ps, the substrate atoms and the chemically bonded Mg atoms vibrate around their equilibrium positions, remaining solid-like. This indicates the high stability of Mg coated layer on the $NbC_{\{001\}}$ surface in liquid Mg.

Supplementary references

s1. Atiemo-Obeng, V. A. & Calabrese, R. V. Rotor–stator mixing devices. *Handbook of industrial mixing: Science and practice*, 479–505.

s2. Kelly, A. & Tyson, W. R. Tensile properties of fibre-reinforced metals: Copper/tungsten and copper/molybdenum. *J. Mech. Phys. Solids* **13**, 329–350 (1965).

s3. Clyne, T. W. & Withers, P. J. in *An introduction to metal matrix composites* (Cambridge university press, 1993).

s4. Taya, M. & Arsenault, R. J. in *Metal matrix composites: thermomechanical behavior* (Elsevier, 2016).

s5. Withers, P. J. & Clarke, A. P. A neutron diffraction study of load partitioning in continuous Ti/SiC composites. *Acta Materialia* **46**, 6585–6598 (1998).

s6. Park, K. & Paulino, G. H. Cohesive zone models: a critical review of traction-separation relationships across fracture surfaces. *Appl. Mech. Rev.* **64**, 060802 (2011).

s7. Li, H. *et al*. Microstructure and properties of carbon nanotubes-reinforced magnesium matrix composites fabricated via novel in situ synthesis process. *J. Alloys Compounds* **785**, 146–155 (2019).

s8. Rashad, M., Pan, F. S., Asif, M. & Ullah, A. Improved mechanical properties of magnesium–graphene composites with copper–graphene hybrids. *Materials Science and Technology* **31**, 1452–1461 (2015).

s9. Nai, M. H., Wei, J. & Gupta, M. Interface tailoring to enhance mechanical properties of carbon nanotube reinforced magnesium composites. *Mater Des* **60**, 490–495 (2014).

s10. Wu, L. *et al*. Microstructure and Mechanical Properties of CNT-Reinforced AZ31 Matrix Composites Prepared Using Hot-Press Sintering. *Journal of Materials Engineering and Performance* **26**, 5495–5500 (2017).

s11. Dinaharan, I., Zhang, S., Chen, G. & Shi, Q. Titanium particulate reinforced AZ31 magnesium matrix composites with improved ductility prepared using friction stir processing. *Materials Science and Engineering: A* **772**, 138793 (2020).

s12. Rashad, M. *et al*. High temperature formability of graphene nanoplatelets-AZ31 composites fabricated by stir-casting method. *Journal of Magnesium and Alloys* **4**, 270–277 (2016).

s13. Yu, W. *et al*. Microstructure, mechanical properties and fracture mechanism of Ti_2AlC reinforced AZ91D composites fabricated by stir casting. *J. Alloys Compounds* **702**, 199–208 (2017).

s14. Khandelwal, A., Mani, K., Srivastava, N., Gupta, R. & Chaudhari, G. P. Mechanical behavior of AZ31/Al_2O_3 magnesium alloy nanocomposites prepared using ultrasound assisted stir casting. *Composites Part B: Engineering* **123**, 64–73 (2017).

s15. Wang, X. J. *et al*. Processing, microstructure and mechanical properties of micro-SiC particles reinforced magnesium matrix composites fabricated by stir casting assisted by ultrasonic treatment processing. *Mater Des* **57**, 638–645 (2014).

s16. Shimizu, Y. *et al*. Multi-walled carbon nanotube-reinforced magnesium alloy composites. *Scr. Mater.* **58**, 267–270 (2008).

s17. Kondoh, K. *et al*. Microstructural and mechanical analysis of carbon nanotube reinforced magnesium alloy powder composites. *Materials Science and Engineering: A* **527**, 4103–4108 (2010).

s18. Habibi, M. K., Joshi, S. P. & Gupta, M. Hierarchical magnesium nano-composites for enhanced mechanical response. *Acta Materialia* **58**, 6104–6114 (2010).

s19. Wang, L. & Chen, T. Simultaneously enhancing strength and toughness of graphene oxide reinforced ZK60 magnesium matrix composites through powder thixoforming. *Composites Part A: Applied Science and Manufacturing* **161**, 107097 (2022).

s20. Zhang, Y. *et al*. Regulating the bimodal structure and strength-ductility synergy of Zn-decorated Ti particles reinforced AZ91 composite through high-volume fraction $Mg_{17}Al_{12}$ precipitations. *Composites Part A: Applied Science and Manufacturing* **185**, 108376 (2024).

s21. Rong, X. *et al*. Revealing the strengthening and toughening mechanisms of Al-CuO composite fabricated via in-situ solid-state reaction. *Acta Materialia* **204**, 116524 (2021).

s22. Slipenyuk, A., Kuprin, V., Milman, Y., Goncharuk, V. & Eckert, J. Properties of P/M processed particle reinforced metal matrix composites specified by reinforcement concentration and matrix-to-reinforcement particle size ratio. *Acta Materialia* **54**, 157–166 (2006).

s23. Knowles, A. J., Jiang, X., Galano, M. & Audebert, F. Microstructure and mechanical properties of 6061 Al alloy based composites with SiC nanoparticles. *J. Alloys Compounds* **615**, S401–S405 (2014).

s24. Dan, C. *et al*. Achieving ultrahigh fatigue resistance in AlSi10Mg alloy by additive manufacturing. *Nature Materials* **22**, 1182–1188 (2023).

s25. Fu, S. *et al*. Study on the microstructure and mechanical properties of selective laser melted TiB/Ti6Al4V composites incorporating trace amounts of TiB_2 nanoparticles. *J. Alloys Compounds* **1013**, 178485 (2025).

s26. Huang, L., Wang, L., Qian, M. & Zou, J. High tensile-strength and ductile titanium matrix composites strengthened by TiB nanowires. *Scr. Mater.* **141**, 133–137 (2017).

s27. Zhou, Z., Liu, Y., Liu, X., Zhan, Q. & Wang, K. Microstructure evolution and mechanical properties of in-situ Ti6Al4V-TiB composites manufactured by selective laser melting. *Composites Part B: Engineering* **207**, 108567 (2021).

s28. Zhou, Q. *et al*. The distribution of reinforcements in titanium matrix composites enhanced with graphene: From dispersed to networked. *Carbon* **226**, 119204 (2024).

s29. Yan, Q. *et al*. Improved mechanical properties in titanium matrix composites reinforced with quasi-continuously networked graphene nanosheets and in-situ formed carbides. *Journal of Materials Science & Technology* **96**, 85–93 (2022).

s30. Liu, Z. *et al*. High-performance titanium-based composite strengthened with in-situ network-distributed 3D reinforcements. *Materials Science and Engineering: A* **802**, 140572 (2021).

s31. Liu, Y. *et al*. Microstructure and mechanical properties of SiC nanowires reinforced titanium matrix composites. *J. Alloys Compounds* **819**, 152953 (2020).

s32. Shang, C., Zhang, F., Zhang, B. & Chen, F. Interface microstructure and strengthening mechanisms of multilayer graphene reinforced titanium alloy matrix nanocomposites with network architectures. *Mater Des* **196**, 109119 (2020).

s33. Wang, Y. *et al*. In-situ synthesized TiC/Ti-6Al-4V composites by elemental powder mixing and spark plasma sintering: Microstructural evolution and mechanical properties. *J. Alloys Compounds* **947**, 169557 (2023).

s34. Yan, Q. *et al*. Formation of dual quasi-continuous networked structure and its strengthening effect in Ti-6Al-4V alloy reinforced with graphene via powder bed fusion. *Additive Manufacturing* **92**, 104364 (2024).

s35. Pan, D. *et al*. Enhanced strength and ductility of nano-TiB_w-reinforced titanium matrix composites fabricated by electron beam powder bed fusion using Ti6Al4V–TiB_w composite powder. *Additive Manufacturing* **50**, 102519 (2022).

s36. Jiao, Y. *et al*. Strengthening and plasticity improvement mechanisms of titanium matrix composites with two-scale network microstructure. *Powder Technol* **356**, 980–989 (2019).

s37. Emura, S., Hagiwara, M. & Yang, S. J. Room-temperature tensile and high-cycle-fatigue strength of fine TiB particulate-reinforced Ti-22Al-27Nb composites. *Metallurgical and Materials Transactions A* **35**, 2971–2979 (2004).

s38. Chen, W. *et al*. Additive manufacturing of high-performance 15-5PH stainless steel matrix composites. *Virtual and Physical Prototyping* **17**, 366–381 (2022).

s39. Diler, E. A. A modified model for the prediction of yield strength of nano-ZrO_2 particle-reinforced austenitic steel matrix nanocomposites. *Measurement* **180**, 109299 (2021).

s40. Ozsoy, A., Aydogan, E. & Dericioglu, A. F. Selective laser melting of Nano-TiN reinforced 17-4 PH stainless steel: Densification, microstructure and mechanical properties. *Materials Science and Engineering: A* **836**, 142574 (2022).

s41. Oliver, W. C. & Pharr, G. M. An improved technique for determining hardness and elastic modulus using load and displacement sensing indentation experiments. *J. Mater. Res.* **7**, 1564–1583 (1992).
