# Supplementary material for: Ultrahigh strength magnesium via solidification of nanocolloid
Source: Nat Commun. 2026 Apr 10;17:5106. doi: 10.1038/s41467-026-71671-x (PMC13246850; doi:10.1038/s41467-026-71671-x)
Supplement: Supplementary file 2 — Description of Additional Supplementary Files [file 41467_2026_71671_MOESM2_ESM.pdf]

Description of Additional Supplementary Files

File Name: Supplementary Video 1

Description: Micro-tensile testing of the Mg-$NbC_{submicron}$ material.

File Name: Supplementary Video 2

Description: Micro-tensile testing of the Mg-NbC_{nano} material.

File Name: Supplementary Video 3

Description: Micro-tensile testing of the reference Mg.
