# Supplementary material for: Ultrahigh strength magnesium via solidification of nanocolloid
Source: Nat Commun. 2026 Apr 10;17:5106. doi: 10.1038/s41467-026-71671-x (PMC13246850; doi:10.1038/s41467-026-71671-x)
Supplement: Supplementary file 6 — Transparent Peer Review file [file 41467_2026_71671_MOESM6_ESM.pdf]

nature portfolio

Peer Review File

Ultrahigh strength magnesium via solidification of nanocolloid

Corresponding Author: Professor Hari Babu Nadendla

This file contains all reviewer reports in order by version, followed by all author rebuttals in order by version.

Version 0:

Reviewer comments:

Reviewer #1

(Remarks to the Author)
The manuscript titled "Ultrahigh strength magnesium via solidification of nanocolloid" presents a comprehensive study on Mg–NbC composites produced using NbC particles of varying sizes (nano, submicron, and micron). The work combines solidification experiments, detailed electron microscopy, and ab initio molecular dynamics simulations to investigate colloidal stability and interfacial bonding. The technical depth and breadth of data presented, particularly in the Supplementary Information, are impressive.

However, some critical issues need clarification:

1. The manuscript repeatedly compares the strengthenability of composites reinforced with different NbC particle sizes, but this term is not rigorously defined. The key experimental comparison should focus on how the absolute mechanical properties—yield strength, ductility, and modulus—evolve with particle size and volume fraction. If "strengthenability" refers to strengthening efficiency, the authors must clearly define the normalization basis (e.g., per particle surface area, interface volume, or particle-matrix load transfer coefficient).

2. Given that the authors invoke Mg@NbC coating and CNbC-like interfacial layers, the particle-size dependence should naturally be linked to surface-to-volume ratio and interfacial energetics. Yet, no direct evidence or quantitative discussion of how surface energy or bonding characteristics evolve with particle size is provided. A clear correlation between AIMD-derived interface stability and experimental mechanical performance is missing.

3. Table S2 shows that the NbC volume fraction increases dramatically with particle size—from 12% (nano) to ~54% (micron). This large variation introduces a confounding factor: it is unclear whether the observed strengthening trend arises primarily from particle size, particle fraction, or both. A normalization or controlled comparison (e.g., constant volume fraction) would help isolate the dominant strengthening mechanism.

4. The manuscript attributes strength enhancement to size effects and interface ordering, yet contributions from grain refinement, load transfer, and dislocation–particle interactions are not separated. Clarification through composite modeling or a rule-of-mixtures comparison would enhance the credibility of the claims.

5. The paper is data-rich but difficult to navigate. The key mechanistic insights—particularly the transition from colloidal stability to interface-controlled strengthening—should be summarized more clearly within the main text rather than being deferred to supplementary sections.

Overall, this is a technically strong and potentially impactful study, but the mechanistic rationale linking particle size, interface stability, and mechanical strengthening can be improved.

Reviewer #2

(Remarks to the Author)

This paper presents an innovative approach to producing ultrahigh-strength magnesium by infiltrating molten Mg into a pellet of NbC nanoparticles. It combined atomic-scale experimental evidence and first-principles DFT simulations to demonstrate the spontaneous wetting and coherent bonding at the Mg/NbC interface. The fabrication method is fairly straight forward. A uniform dispersion of NbC nanoparticles without relying on external fields or complex thermomechanical processing was achieved.

However, the powder pellet method is somewhat difficult to scale up for large scale production. The pellet method may limit its flexibility of NbC particle volume percent. Long-term thermal stability of the Mg-NbC composites will be of interest.

Overall, it is a plausible paper with merit.

Reviewer #3

(Remarks to the Author)
The authors have undertaken a very interesting study on the role of dispersed nano-particles on the properties of Mg, which they are calling a nano-colloid. This has been the challenge with nano-composites, i.e. how to achieve a homogeneous dispersion of nano-particles, rather than finding them in clusters. I am aware of such processes having been used previously to create nano-composites. Some of the challenge can be when these pellets are added to melts particularly with gravity separation because normally lower levels of particles are preferred.

One of the drawbacks of this paper is that it compares the properties of these materials with standard alloys. However, what is shown here are metal matrix compositions with a very large amount of the second phase. The volume % are 12, 39 and 54%, however it should be realised that given the density different the mass% is a lot greater. This is the primary reason for the increase in modulus and hardness, i.e. that is it not really a Mg based material, it is primarily a ceramic. Having said that the nano-particle version still is majority Mg although it is still high enough content to be a metal matrix composite and should be compared with other MMCs. So comparison with similar materials is required.

I also do not believe that the micro-tensile tests are providing a useful comparison of the properties. Samples at this size are well below critical flaw sizes and consequently do not give a reasonable estimate of the tensile properties that can be expected in typical use cases. Nor should these results be compared with results from macro-tensile tests. The failure is completely different as shown by the tensile curve results. The failure is seen in Supp Fig 7 which is interesting but not helpful for comparision with bulk properties. The real tensile data is observed in Supplementary Fig 2 which is very different. So comparison of materials in the same or similar test is required.

I think that when appropriate materials and testing methods are compared the properties shown are not as outstanding as it appears.

There is a nice microscopic and DFT study of the interface indicating that there is templating on the particles as has been described previously by researchers from Brunel in this group. Whilst DFT is a useful tool, it is not the only tool to understanding the interface behaviour. The conclusions indicate that DFT can predict the wetting behaviour of metals on ceramic nano-particles. It is not clear to me that NbC was chosen because of the these calculations. If not, I would be interested in seeing results that backed that statement up more strongly.

Line 246 terminal is misspelled.
Supp Fig 8 has misspelled label on y-axis.

Version 1:

Reviewer comments:

Reviewer #1

(Remarks to the Author)
The authors have thoroughly addressed my previous comments, resulting in substantial improvements to the manuscript. The work is now complete and suitable for publication in its current form.

Reviewer #2

(Remarks to the Author)
It seems the authors addressed my comments reasonably well. The dilution of master nanocomposites into magnesium alloys shows good results too.

Reviewer #3

(Remarks to the Author)
I still have a lot of concerns about how the results are presented, particularly with the use of micro-tensile testing. The values should not be compared with other sanples where macro-testing is used. This gives a false impression that the properties are a lot better than they actually are. In SSupplementary Figure 2 we see the Mg14%NbC alloy has a yield strength of

approximately 150MPa with an elongation to fracture of about 1%. This is typical for alloys such as this one. This is very different from the 678MPa yield strength improvement mentioned in the manuscript. If this is only compared with other micro tensile tests that would be fine, but that does not seem to be the case.

Version 2:

Reviewer comments:

Reviewer #3

(Remarks to the Author)
Whilst this is an improvement, I request that the authors specifically say that the tensile results are from micro-tensile tests within the caption in Fig 2a. That is:
a. 'Micro'-tensile stress-strain curves

This will make it clear to the readers what they are looking at.

Open Access This Peer Review File is licensed under a Creative Commons Attribution 4.0 International License, which permits use, sharing, adaptation, distribution and reproduction in any medium or format, as long as you give appropriate credit to the original author(s) and the source, provide a link to the Creative Commons license, and indicate if changes were made.
In cases where reviewers are anonymous, credit should be given to 'Anonymous Referee' and the source.
The images or other third party material in this Peer Review File are included in the article's Creative Commons license, unless indicated otherwise in a credit line to the material. If material is not included in the article's Creative Commons license and your intended use is not permitted by statutory regulation or exceeds the permitted use, you will need to obtain permission directly from the copyright holder.
To view a copy of this license, visit https://creativecommons.org/licenses/by/4.0/

Authors response to reviewers' comments (NCOMMS-25-78483-T)

Reviewer #1 *(Remarks to the Author):*
The manuscript titled "Ultrahigh strength magnesium via solidification of nanocolloid" presents a comprehensive study on Mg–NbC composites produced using NbC particles of varying sizes (nano, submicron, and micron). The work combines solidification experiments, detailed electron microscopy, and ab initio molecular dynamics simulations to investigate colloidal stability and interfacial bonding. The technical depth and breadth of data presented, particularly in the Supplementary Information, are impressive.

Overall, this is a technically strong and potentially impactful study, but the mechanistic rationale linking particle size, interface stability, and mechanical strengthening can be improved.

We thank reviewer for identifying the strengths and seeking further clarification on the following points:

R1.1*. The manuscript repeatedly compares the strengthenability of composites reinforced with different NbC particle sizes, but this term is not rigorously defined. The key experimental comparison should focus on how the absolute mechanical properties—yield strength, ductility, and modulus—evolve with particle size and volume fraction. If "strengthenability" refers to strengthening efficiency, the authors must clearly define the normalization basis (e.g., per particle surface area, interface volume, or particle-matrix load transfer coefficient).*

Response: We thank the reviewer for highlighting the ambiguity associated with the term "strengthening effectiveness (strengthenability)" in the original manuscript. We agree that, without a rigorous definition and clear normalisation basis, this terminology could lead to confusion when comparing composites reinforced with different particle sizes and volume fractions.

In the revised manuscript, we have therefore removed the use of the term *"strengthening effectiveness (strengthenability)"*. Instead, the discussion now focuses explicitly on the absolute evolution of mechanical properties, particularly the yield-strength increment, $\Delta\sigma_{YS}$ as a function of NbC particle size and volume fraction (Fig. 2**b**). This approach provides a direct and unambiguous basis for comparison, consistent with the reviewer's recommendation.

To clearly separate the effects of particle size and volume fraction on yield strength enhancement, we have added a new plot (Fig. 2**b**) showing $\Delta\sigma_{YS}$ as a function of NbC volume fraction for all particle sizes investigated. In addition, the individual strengthening contributions arising from particle-dislocation interactions (Orowan strengthening) and load transfer are explicitly evaluated using particle size dependent parameters. The governing expressions for these mechanisms are detailed in the Methods section, with an expanded discussion of the load-transfer mechanism provided in Supplementary Note 2.

By presenting both the experimentally measured yield strength increments and the corresponding mechanistic contributions as functions of particle size and volume fraction, the revised manuscript provides a physically meaningful and consistent framework for comparing strengthening behaviour, without reliance on ambiguous terminology. Accordingly,

the statement in the original manuscript reporting specific strengthening effectiveness in unit of MPa/vol% for different particle sizes has been removed.

***R1.2**. Given that the authors invoke Mg@NbC coating and CNbC-like interfacial layers, the particle-size dependence should naturally be linked to surface-to-volume ratio and interfacial energetics. Yet, no direct evidence or quantitative discussion of how surface energy or bonding characteristics evolve with particle size is provided. A clear correlation between AIMD-derived interface stability and experimental mechanical performance is missing.*

Response: We agree with the reviewer that the particle-size dependence of strengthening should be quantitatively linked to surface-to-volume effects and interfacial energetics and further connected to a physically meaningful strengthening framework. In the revised manuscript, we now explicitly connect AIMD-derived Mg-C bonding, particle-size-dependent interfacial atom fraction, interface-limited debonding strength, and load-transfer strengthening to the experimentally observed mechanical response (Supplementary Note 2).

Ab initio molecular dynamics simulations reveal strong chemical bonding between superficial C atoms on NbC and terminating Mg atoms, forming ordered Mg-C interfacial bonds (Fig. 4). These predictions are consistent with STEM observations of an interfacial multilayer complexion that is epitaxially coherent with the NbC surface (Fig. 3), indicating the formation of Mg-coated NbC particles (Mg@NbC) in the liquid Mg that are retained upon solidification. The AIMD calculations further yield an interfacial fracture energy of $\Gamma = 1.54$ J·m^{-2}, providing a quantitative metric of interface stability.

To translate this interfacial energetics into a mechanical limit for stress transfer, we evaluated the interface-limited debonding strength using a fracture-energy-based criterion for a stiff spherical inclusion embedded in a compliant matrix[s6]:

$$\sigma_{\mathrm{debond}}(d) = \frac{1}{3(1-\nu_m)}\sqrt{\frac{16\,\Gamma\,E_m(1+\nu_m)}{d}}$$

where $E_m$and $\nu_m$are the Young's modulus and Poisson's ratio of Mg. Using $E_m = 45$GPa, $\nu_m = 0.35$, and $\Gamma = 1.54$ J·m^{-2}, we obtain predicted debonding stresses of approximately 5.12 GPa (15 nm), 1.17 GPa (287 nm), and 0.66 GPa (906 nm). These gigapascal-level values indicate that the Mg-NbC interface is sufficiently strong to sustain stress transfer across all particle sizes studied, with increasing resistance to debonding as particle size decreases.

In parallel, the particle-size dependence of the population of chemically bonded interfacial atoms was estimated by considering the ratio of surface to bulk C atoms in a cubic NbC particle with {001} surfaces:

$$R = \frac{n(C_{\mathrm{surf}})}{n(C_{\mathrm{bulk}})} = \frac{6d^2/a^2}{4d^3/a^3} = \frac{1.5}{d/a}$$

where $a = 4.469$ Å is the NbC lattice parameter and d is the particle size. This scaling shows that the fraction of surface atoms decreases rapidly with increasing particle size, providing a quantitative explanation for why interfacial chemistry is expected to contribute most strongly at the nanoscale.

Finally, we connect these interfacial considerations to load transfer strengthening. Under the iso-strain (Voigt) load-sharing condition appropriate for well-bonded particulate composites, the load transfer strengthening contribution[s4, s5] is:

$$\Delta\sigma_{\mathrm{LT,Voigt}} = (\frac{E_p}{E_m} - 1)V_p\sigma_{YS}^m$$

where E_p is the NbC modulus, V_p is the particle volume fraction, and σ_{YS}^m is the yield strength of the unreinforced Mg matrix processed under identical conditions ($\sigma_{YS}^m = 35$ MPa). Using representative moduli $E_p/E_m \approx 10$, this reduces to $\Delta\sigma_{\mathrm{LT,Voigt}} = 9V_p\sigma_{YS}^m$. For the three compositions corresponding to the particle sizes studied here, the predicted load transfer contributions are:

- 15 nm, 12.2 vol.% NbC: $\Delta\sigma_{\mathrm{LT}} = 9(0.122)(35) \approx 38.4$MPa
- 287 nm, 38.7 vol.% NbC: $\Delta\sigma_{\mathrm{LT}} = 9(0.387)(35) \approx 121.9$MPa
- 906 nm, 13 vol.% NbC: $\Delta\sigma_{\mathrm{LT}} = 9(0.13)(35) \approx 41.0$MPa

Crucially, the interface must be able to sustain these stress-transfer demands. A conservative interface-capacity estimate may be written as $\Delta\sigma_{\mathrm{LT,max}} \sim \eta V_p \sigma_{\mathrm{debond}}(d)$with $\eta \approx 0.5$. Using the above σ_{debond}values, the interface-limited capacities remain far above the Voigt load transfer requirements for all three cases, confirming that the Mg-NbC system operates in the perfect load transfer (Voigt-limited) regime. Therefore, for the present Mg-NbC materials, particle size does not limit load transfer through debonding; instead, particle size primarily modulates the relative volumetric significance of interfacial chemistry (*via* the interfacial atom fraction R), while the overall macroscopic strengthening is governed by the combined effects of particle fraction (load transfer) and dislocation-mediated Orowan mechanisms.

The detailed load transfer increment ($\Delta\sigma_{\mathrm{LT}}$) assessment considering both the interface-limited debonding and Voigt load-sharing mechanisms are presented in Supplementary Note 2.

References

s4. Taya, M. & Arsenault, R. J. in *Metal matrix composites: thermomechanical behavior* (Elsevier, 2016).
s5. Withers, P. J. & Clarke, A. P. A neutron diffraction study of load partitioning in continuous Ti/SiC composites. *Acta Materialia* **46**, 6585–6598 (1998).
s6. Park, K. & Paulino, G. H. Cohesive zone models: a critical review of traction-separation relationships across fracture surfaces. *Appl. Mech. Rev.* **64**, 060802 (2011).

***R1.3**. Table S2 shows that the NbC volume fraction increases dramatically with particle size—from 12% (nano) to ~54% (micron). This large variation introduces a confounding factor: it is unclear whether the observed strengthening trend arises primarily from particle size, particle fraction, or both. A normalization or controlled comparison (e.g., constant volume fraction) would help isolate the dominant strengthening mechanism.*

Response: We agree with the reviewer that the effect of the particle size and particle fraction should be explicitly compared to understand the participating strengthening mechanisms. To address this, we have added Fig. 2**b**, which presents the strengthening contribution as a function of NbC volume fraction for all three particle sizes studied. This normalisation allows the effect of particle size to be separated from that of particle fraction. For example, at a volume fraction of approximately 13%, the calculations indicate that smaller particle sizes correspond to higher yield-strength increments. This trend is exemplified by comparing the Mg-NbC_{nano} (12.2 vol.%) and Mg-NbC_{micron} (13 vol.%) samples,

which exhibit similar predicted load-transfer contributions (see the response to ***R1.2*** above) but markedly different Orowan contributions due to the particle-size effect (see the response to ***R1.4*** below).

Furthermore, in the inset to Fig. 2**b**, we now compare the yield strength improvement of the present Mg-NbC materials with a wide range of metal matrix composites produced by melt processing, powder metallurgy, and additive manufacturing routes. This contextual comparison highlights the exceptional strengthening contribution achieved via the colloidal solidification approach.

R1.4*. The manuscript attributes strength enhancement to size effects and interface ordering, yet contributions from grain refinement, load transfer, and dislocation–particle interactions are not separated. Clarification through composite modelling or a rule-of-mixtures comparison would enhance the credibility of the claims.*

Response: Grain refinement can be excluded for the micro-tensile test as the length scale of sample is ~10 μm, while the corresponding Mg grain size of the same sample is > 150 μm. The dominant strengthening mechanisms in this work are therefore particle-dislocation interactions (Orowan strengthening) and load transfer. The Orowan contribution is now explicitly calculated using established models and is presented in Fig. R1 as a function of NbC volume fraction for all particle sizes.

The load-transfer contribution, originating from stress transfer across the Mg-NbC interface, is calculated using a composite mechanics framework and is presented in Fig. R2. By combining the contributions shown in Figs. R1 and R2, the theoretical prediction of the yield strength improvement of the Mg-NbC system is obtained and presented in Fig. 2**b** in the revised manuscript.

A section named “Strengthening contribution calculations” section is now added to the Methods in the revised manuscript. It includes the details of the Orowan strengthening and load transfer strengthening calculations.

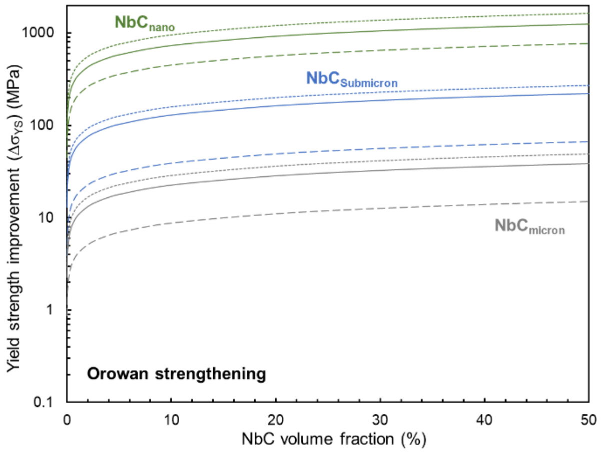

Figure R1. Calculated yield strength improvement ($\Delta\sigma_{YS}$) of Mg-NbC materials via Orowan strengthening with nano, submicron, and micron sized particles. The dotted, solid and dashed lines correspond to the calculation with $d_{peak-\sigma}$, d_{peak}, and $d_{peak+\sigma}$ particle size.

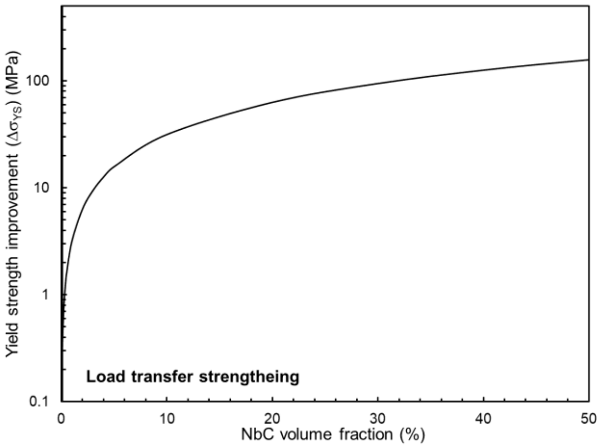

Figure R2. Calculated yield strength improvement ($\Delta\sigma_{YS}$) of Mg-NbC materials via Load transfer strengthening as a function of the particle volume fraction.

***R1.5**. The paper is data-rich but difficult to navigate. The key mechanistic insights—particularly the transition from colloidal stability to interface-controlled strengthening—should be summarized more clearly within the main text rather than being deferred to supplementary sections.*

Response: We have reorganised the manuscript accordingly. The discussion of colloidal stability, which is critical for achieving uniform dispersion during solidification, has been moved from the Supplementary Information to the main text in the revised manuscript. This is seen in the section “Stable Mg-NbC nanocolloid at high temperature” on page 13.

Reviewer #2 (Remarks to the Author):
This paper presents an innovative approach to producing ultrahigh-strength magnesium by infiltrating molten Mg into a pellet of NbC nanoparticles. It combined atomic-scale experimental evidence and first-principles DFT simulations to demonstrate the spontaneous wetting and coherent bonding at the Mg/NbC interface. The fabrication method is fairly straight forward. A uniform dispersion of NbC nanoparticles without relying on external fields or complex thermomechanical processing was achieved.

Overall, it is a plausible paper with merit

We thank the reviewer for their positive assessment of the manuscript and for recognising the novelty of the processing strategy, the atomic-scale experimental evidence, and the integration of first-principles simulations. We also appreciate the reviewer's constructive comments regarding scalability and thermal stability, which we address below:

***R2.1**. The powder pellet method is somewhat difficult to scale up for large scale production. The pellet method may limit its flexibility of NbC particle volume percent.*

Response: We appreciate this important point. The original motivation for developing the pellet infiltration process was to enable the safe and controlled introduction of nanoparticles into molten Mg, particularly in industrial environments where direct handling of nano powders is challenging. Importantly, the infiltration process (Fig. 1) produces a highly concentrated Mg-NbC master alloy, rather than a final-composition material.

This master alloy can subsequently be diluted to any desired particle volume fraction using conventional melt processing, making the approach compatible with standard foundry practice. In the revised manuscript, we now explicitly demonstrate this concept by reporting the dilution of a concentrated Mg-NbC$_{submicron}$ master alloy to produce bulk material with 1 vol.% NbC particle loading. Corresponding macroscopic billet, tensile specimens, and stress-strain curves are now included (Supplementary Fig. 7), demonstrating both scalability and compositional flexibility of the process.

***R2.2**. Long-term thermal stability of the Mg-NbC composites will be of interest.*

Response: We agree with the reviewer that long-term thermal stability is an important consideration. One anticipated advantage of the present Mg-NbC system is the use of a thermally stable ceramic phase, in contrast to conventional Mg alloys where strengthening relies on temperature-sensitive precipitates. While a comprehensive high-temperature mechanical and microstructural stability study is beyond the scope of the present manuscript, such investigations are currently ongoing and will be reported in a future dedicated publication once completed. This aspect has been included in this revised manuscript.

Reviewer #3 (Remarks to the Author):
The authors have undertaken a very interesting study on the role of dispersed nano-particles on the properties of Mg, which they are calling a nano-colloid. This has been the challenge with nano-composites, i.e. how to achieve a homogeneous dispersion of nano-particles, rather than finding them in clusters. I am aware of such processes having been used previously to create nano-composites. Some of the challenge can be when these pellets are added to melts particularly with gravity separation because normally lower levels of particles are preferred.

We thank the reviewer for the thoughtful and constructive assessment of our work, and for recognising the long-standing challenge of achieving homogeneous dispersion of nanoparticles in metal matrix composites. We appreciate the opportunity to clarify the scope, comparisons, and interpretation of our results.

In the present work, in addition high volume particle loading, we also demonstrate that homogeneous dispersion at lower particle loading (~1 vol.% $NbC_{submicron}$) can be achieved using an infiltrated Mg-NbC master pellet (Supplementary Fig.7), which is introduced into the melt under controlled processing conditions. This approach mitigates particle clustering by preventing direct particle-particle contact during addition and promotes uniform distribution through wetting-assisted dispersion and melt convection. Furthermore, the discussion in "*Assessment of colloid stability for larger particles*" in Supplementary Note 1 addresses the stability of Mg-NbC colloid in the presence of gravity.

R3.1. *One of the drawbacks of this paper is that it compares the properties of these materials with standard alloys. However, what is shown here are metal matrix compositions with a very large amount of the second phase. The volume % are 12, 39 and 54%, however it should be realised that given the density different the mass% is a lot greater. This is the primary reason for the increase in modulus and hardness, i.e. that is it not really a Mg based material, it is primarily a ceramic. Having said that the nano-particle version still is majority Mg although it is still high enough content to be a metal matrix composite and should be compared with other MMCs. So comparison with similar materials is required.*

Response: We agree with the reviewer that materials with high reinforcement volume fractions should be compared primarily with other metal matrix composites. To address this point, we have expanded the comparative framework in the revised manuscript.

Specifically, we have collated data from the literature on Mg-, Al-, Ti-, and Fe-based metal matrix composites reinforced with oxides, carbides, borides, and nitrides, which is now presented in Supplementary Tables 1 and 3. A direct comparison between these established MMC systems and the present Mg-NbC materials is provided as inset in Fig. 2**b**. This comparison shows that, even when benchmarked against other MMCs with comparable reinforcement levels, the Mg-NbC system processed using simple solidification process exhibits remarkable improvement in yield strength. The dataset in Fig. 2**d** compares strength and modulus relative to density of a range of alloys and their composites.

R3.2. *I also do not believe that the micro-tensile tests are providing a useful comparison of the properties. Samples at this size are well below critical flaw sizes and consequently do not give a reasonable estimate of the tensile properties that can be expected in typical use cases. Nor should these results be compared with results from macro-tensile tests. The failure is completely different as shown by the tensile curve results. The failure is seen in*

Supp Fig 7 which is interesting but not helpful for comparison with bulk properties. The real tensile data is observed in Supplementary Fig 2 which is very different. So comparison of materials in the same or similar test is required.

I think that when appropriate materials and testing methods are compared the properties shown are not as outstanding as it appears.

Response: We agree that micro-tensile and macro-tensile tests probe different aspects of mechanical behaviour and should be interpreted accordingly. We have clarified this distinction more explicitly in the revised manuscript. In the section "Ultrahigh tensile strength in Mg-NbC materials" on page 6, the Fig.2 has been modified and expanded by adding new results, and the corresponding text is also revised.

In this work, micro-tensile testing (Fig. 2**a**) was intentionally performed on materials with a single-crystal Mg matrix, both without particles and with dispersed NbC particles. This design allows a direct and controlled comparison of the intrinsic strengthening effect of NbC particles on Mg deformation, while eliminating contributions from grain boundaries and, texture. In this context, the micro-tensile results are not intended to represent bulk engineering properties, but rather to reveal the fundamental strengthening capability of the Mg-NbC system at the crystal level.

In contrast, newly added Fig. 2**b** includes two distinct datasets: (i) micro-tensile results obtained from Mg-NbC material (square symbol), and (ii) macro-tensile results obtained from polycrystalline Mg-NbC materials (diamond symbol) containing submicron and micron-sized NbC particles, following ASTM E8 standard. These macro-tensile results reflect bulk mechanical behaviour and incorporate the effects of grain structure, particle distribution, and processing-induced defects.

The two testing approaches are used in a complementary manner (micro-tensile tests establish the intrinsic, upper-bound strengthening potential arising from particle-matrix interactions, while macro-tensile tests demonstrate the translation of this potential to bulk, polycrystalline materials at realistic length scales). The yield strength improvement measured in macro and micro-tensile experiments are consistent with the theoretical predicted tendency for all particle size and volume fraction. Dataset in Fig. 2**b**, provide a coherent picture of mechanical response at both local and bulk Mg-NbC materials.

R3.3. *There is a nice microscopic and DFT study of the interface indicating that there is templating on the particles as has been described previously by researchers from Brunel in this group. Whilst DFT is a useful tool, it is not the only tool to understanding the interface behaviour. The conclusions indicate that DFT can predict the wetting behaviour of metals on ceramic nano-particles. It is not clear to me that NbC was chosen because of the these calculations. If not, I would be interested in seeing results that backed that statement up more strongly.*

Response: We first experimentally observed the exceptional wetting characteristic and colloidal stability of NbC in liquid Mg. The *ab initio* molecular dynamics simulations were subsequently performed to rationalise and interpret this experimentally observed phenomenon at the atomic scale. The simulations reveal the formation of ordered Mg-C interfacial bonding and provide a quantitative measure of interfacial stability, supporting the experimental observations. We believe DFT calculation can help in accelerating ceramic

phase selection for metallic materials by investigating the formation of ordered layer on ceramic phase as one of the necessary conditions to realise the excellent wetting of the molten metal to the *ex-situ* incorporated ceramic phases.

***R3.4**. Line 246 terminal is misspelled.*
Supp Fig 8 has misspelled label on y-axis.

Response: All identified typographical issues have been corrected, and a comprehensive proofreading of the Manuscript and Supplementary Information has been conducted to improve overall clarity and presentation.

Response to Reviewer #3's Comments (NCOMMS-25-78483A)

***Reviewer #3** (Remarks to the Author):*
I still have a lot of concerns about how the results are presented, particularly with the use of micro-tensile testing. The values should not be compared with other sanples where macro-testing is used. This gives a false impression that the properties are a lot better than they actually are. In SSupplementary Figure 2 we see the Mg14%NbC alloy has a yield strength of approximately 150MPa with an elongation to fracture of about 1%. This is typical for alloys such as this one. This is very different from the 678MPa yield strength improvement mentioned in the manuscript. If this is only compared with other micro tensile tests that would be fine, but that does not seem to be the case.

We thank the reviewer for the careful evaluation of our revised manuscript and for raising this important concern regarding the comparison of micro- and macro-tensile data.

We agree that elongation to fracture can depend on specimen size and that direct comparison between micro- and macro-scale tensile tests may lead to misleading interpretation. To address this concern, revised Fig. 2**d** has been added to the revised manuscript. The updated figure now includes only literature data (see Supplementary Table 3) obtained from comparable specimen sizes employing micro-tensile testing. The revised Fig. 2**d** (Mg-NbC versus micro-tensile test data from the literature) shows the same overall trend as that presented in the previous version of the figure.

Figure 2**b** (inset) presents the yield strength increment ($\Delta\sigma_{YS}$) between the monolithic alloy and the corresponding reinforced alloy, where each comparison is made using the same testing methodology within the respective study. Therefore, the comparison is methodologically consistent and does not involve mixing micro- and macro-tensile data within a single $\Delta\sigma_{YS}$ calculation. Furthermore, yield strength is determined from the initial plastic deformation region and is not influenced by early fracture behaviour. The load cells used in both micro- and macro-tensile testing systems are calibrated, ensuring reliable force measurement and, consequently accurate stress determination. Therefore, the reported $\Delta\sigma_{YS}$ values reflect the intrinsic strengthening contribution of the reinforcement under the specific testing conditions. For these reasons, Fig. 2**b** (inset) does not create a misleading impression and has been retained without modification.

Response to Reviewer #3's Comments (NCOMMS-25-78483B)

Reviewer #3 *(Remarks to the Author):*
Whilst this is an improvement, I request that the authors specifically say that the tensile results are from micro-tensile tests within the caption in Fig 2a. That is:
a. 'Micro'-tensile stress-strain curves
This will make it clear to the readers what they are looking at.

Response: The word "micro" has been added to the caption of Fig. 2**a**, as suggested by the reviewer, to clarify that the tensile results are obtained from micro-tensile tests.
